# IFN-γ-Stimulated Neutrophils Suppress Lymphocyte Proliferation through Expression of PD-L1

**DOI:** 10.1371/journal.pone.0072249

**Published:** 2013-08-28

**Authors:** Stan de Kleijn, Jeroen D. Langereis, Jenneke Leentjens, Matthijs Kox, Mihai G. Netea, Leo Koenderman, Gerben Ferwerda, Peter Pickkers, Peter W. M. Hermans

**Affiliations:** 1 Laboratory of Pediatric Infectious Diseases, Radboud University Medical Centre, Nijmegen, The Netherlands; 2 Department of Intensive Care Medicine, Radboud University Medical Centre, Nijmegen, The Netherlands; 3 Department of Internal Medicine, Radboud University Medical Centre, Nijmegen, The Netherlands; 4 Department of Anesthesiology, Radboud University Medical Centre, Nijmegen, The Netherlands; 5 Department of Respiratory Medicine, University Medical Center Utrecht, Utrecht, The Netherlands; French National Centre for Scientific Research, France

## Abstract

During systemic inflammation different neutrophil subsets are mobilized to the peripheral blood. These neutrophil subsets can be distinguished from normal circulating neutrophils (CD16^bright^/CD62L^bright^), based on either an immature CD16^dim^/CD62L^bright^ or a CD16^bright^/CD62L^dim^ phenotype. Interestingly, the latter neutrophil subset is known to suppress lymphocyte proliferation *ex vivo*, but how neutrophils become suppressive is unknown. We performed transcriptome analysis on the different neutrophil subsets to identify changes in mRNA expression that are relevant for their functions. Neutrophil subsets were isolated by fluorescence-activated cell sorting from blood of healthy volunteers that were administered a single dose of lipopolysaccharide (2 ng/kg i.v.) and the transcriptome was determined by microarray analysis. Interestingly, the CD16^bright^/CD62L^dim^ suppressive neutrophils showed an interferon-induced transcriptome profile. More importantly, IFN-γ, but not IFN-α or IFN-β stimulated neutrophils, acquired the capacity to suppress lymphocyte proliferation through the expression of programmed death ligand 1 (PD-L1). These data demonstrate that IFN-γ-induced expression of PD-L1 on neutrophils enables suppression of lymphocyte proliferation. Specific stimulation of neutrophils present at the inflammatory sites might therefore have a pivotal role in regulating lymphocyte-mediated inflammation and autoimmune disease.

## Introduction

Neutrophils represent the highest proportion of circulating leukocytes in the peripheral blood. Following invasion of microorganisms, these cells are recruited to the site of infection where they use their antimicrobial capacity to clear invading pathogens [1]. In the last decade, this “conventional” view of neutrophils in the immune response has changed substantially. Besides their capacity to kill invading pathogens, neutrophils have been shown to modulate the immune system on various levels [2]. The first evidence that neutrophils can modulate the response of other immune cells was found in their interaction with dendritic cells (DCs). Neutrophils were shown to induce maturation of monocyte-derived DCs and boost DC cytokine production, resulting in T-cell proliferation and polarization towards a Th1 phenotype [3], [4].

Recent studies have shown that during experimental human endotoxemia i.e. systemic inflammation elicited by LPS administration in healthy volunteers or severe trauma changes the heterogeneity of the circulating neutrophils pool dramatically [5], [6]. Three different neutrophil subsets can be distinguished based on their expression of CD16 and CD62L. CD16^dim^/CD62L^bright^ neutrophils appear to be released from the bone marrow and are characterized by a banded nuclear morphology and immature antimicrobial capacity. CD16^bright^/CD62L^dim^ neutrophils display a hypersegmented nucleus, increased functional antimicrobial capacity and, strikingly, exhibit the capacity to suppress lymphocyte proliferation. This novel immune regulatory mechanism for neutrophils was shown to be dependent on hydrogen peroxide release and expression of integrin MAC-1 (α_M_β_2_) [6]. To date, it is unclear how this CD16^bright^/CD62L^dim^ subset of neutrophils acquires the ability to suppress lymphocyte proliferation. Knowledge on the regulation of this process could have important implications in the modulation of lymphocyte-mediated disease pathology.

Previously, we have shown that the total pool of circulating neutrophils during experimental human endotoxemia has a specific transcriptome profile that was reminiscent to a profile induced by a combination of direct activation by inflammatory cytokines and the influx of young neutrophils from the bone marrow [7]. In the current study, we further investigated the transcriptome of the different neutrophil subsets that emerge in the circulation during experimental human endotoxemia, based on the expression of CD16 and CD62L in order to identify factors involved in generation of suppressive neutrophils. Additionally, we explored the mechanisms behind IFN-γ-induced neutrophil-mediated lymphocyte suppression.

## Materials and Methods

### Subjects and experimental human endotoxemia model

The neutrophil subset transcriptome was studied in 4 healthy male volunteers who participated in a human endotoxemia trial (Clinical Trial Register number NCT01374711, placebo group). The study protocol was approved by the Ethics Committee of the Radboud University Medical Centre and complies with the Declaration of Helsinki including current revisions and the Good Clinical Practice guidelines. Written informed consent was obtained from all study participants.

The experiments were performed according to a strict clinical protocol as described previously [8]. Subjects were screened before the start of the experiment and had a normal physical examination, electrocardiography, and routine laboratory values (including serology on HIV and hepatitis B). Subjects with febrile illness during the two weeks before the experiment were excluded. Subjects were not allowed to take any prescription drugs and asked to refrain from caffeine and alcohol intake 24 hours before the start of the experiment. Furthermore, subjects refrained from food 12 hours before the start of each endotoxemia experiment. After admission to the research intensive care unit of the Radboud University Nijmegen Medical Centre, purified LPS (US Standard Reference Endotoxin *Escherichia Coli O:113)* obtained from the Pharmaceutical Development Section of the National Institutes of Health (Bethesda, MD) was administered at a dose of 2 ng/kg body weight. In all subjects, heart rate (5-lead electrocardiogram) and blood pressure (20-gauge radial artery catheter) were monitored starting 2 hours before administration of LPS until discharge 8 hours after LPS administration. A cannula was placed in an antecubital vein to permit infusion of prehydration fluid (1.5 L 2.5% glucose/0.45% saline 1 hour before LPS administration), endotoxin, and continuous infusion of 2.5% glucose/0.45% saline (150 mL/hour during 8 hours after LPS administration) to ensure optimal hydration status. Body temperature was measured using an infrared tympanic thermometer (FirstTemp Genius, Sherwood Medical, Crawley/Sussex, UK). The course of endotoxin-induced flu-like symptoms (headache, nausea, shivering, and muscle and back pain) was scored every 30 minutes on a 6-point Likert scale (0 = no symptoms, 5 = very severe symptoms), resulting in a total score of 0 to 25.

### FACS analysis

During human endotoxemia experiments, sodium heparin anticoagulated blood was drawn from the arterial line. Erythrocytes were lysed in isotonic ice-cold NH_4_Cl solution (8.3 g/L NH_4_Cl, 1 g/L KHCO_3_ and 37 mg/L EDTA) followed by centrifugation at 4°C. Total leukocytes were washed with PBS and stained with αCD62L, αCD16 and αCD14 (BD Biosciences) for 30 minutes at 4°C. Subsequently, the cells were washed and sorted on the FACSAria II (BD Biosciences). Sorted cell fractions were washed with PBS and dissolved in RLT lysis buffer containing 1% β-mercaptoethanol and immediately frozen at −80°.

### RNA isolation and microarray analysis

RNA was isolated by Qiagen RNAeasy RNA isolation kit according to the manufacturer's protocol. In addition, DNA contamination was removed by on column DNase treatment (Qiagen). Total RNA yield was determined on the nanodrop ND-1000 (Isogen life sciences), and total RNA quality was assessed by the use of RNA 6000 Nano chips on the Agilent 2100 bioanalyzer (Agilent). Neutrophil gene expression was measured on Affymetrix Human ST 1.0 exon arrays. RNA material was first amplified, transformed to cDNA and labeled using ambion WT expression kit and the Affymetrix terminal labeling kit. Labeled cDNA was then hybridized for 17 hours at 42°C to a Human ST 1.0 exon array, washed and stained according to manufacturers' instructions and scanned on a Genechip scanner 3000 (Affymetrix). Microarray data has been made available to the Gene expression omnibus (GEO) with accession number GSE42358.

Affymetrix CEL-files from microarray scans were uploaded in the exon array analyzer tool [9]. After quality control, this tool uses Robust Multiarray Averaging (RMA) analysis for normalization of intensity values and a LIMA statistical analysis for large data sets to determine statistically significant differentially expressed genes in the different groups. The experiment groups at t = 4 hours after LPS were either compared relative to t = 0 hours or compared mutually.

### Neutrophil and PBMC isolation

After written informed consent, blood was drawn from healthy donors in EDTA anticoagulation tubes. Blood was diluted 2∶1 with PBS. Mononuclear cells and granulocytes were separated by centrifugation using Ficoll-Paque. Erythrocytes were lysed in isotonic ice-cold NH_4_Cl solution (8.3 g/L NH_4_Cl, 1 g/L KHCO_3_ and 37 mg/L EDTA) followed by centrifugation at 4°C as described previously [10]. After isolation, granulocytes (>95% pure with eosinophils as major contaminant) were washed in PBS and resuspended in HEPES buffered RPMI 1640 supplemented with 10% FCS. After Ficoll-Paque centrifugation, PBMCs fraction was washed twice with PBS and resuspended in RPMI supplemented with 2 mM L-Glutamine and 10% heat-inactivated human serum.

### CD274, CD273 and CD279 expression experiments

Neutrophils were suspended in HEPES-buffered RPMI supplemented with 10% FCS to a concentration of 5.10^6^/mL and stimulated with 100 ng/mL TNF-α (BD Biosciences), 50 ng/mL G-CSF (R&D systems), 50 ng/mL GM-CSF (Sanquin), 200 ng/mL IFN-α2 (Roche), 100 U/mL IFN-β1A (Invitrogen), 1 to 1000 ng/mL IFN-γ (Sigma), 50 ng/mL LPS (Invitrogen) for 18–20 hours at 37°C+5% CO_2_. For short IFN-γ exposure, neutrophils were stimulated 15 minutes or 2 hours, where after IFN-γ was washed away and neutrophils were further incubated in HEPES-buffered RPMI supplemented with 10% FCS till 18–20 hours at 37°C+5% CO_2_. For kinetic experiments, neutrophils were stimulated 0, 2, 4, 6, 8 or 20 hours. Neutrophils were washed once and stained for αCD274, αCD273 or αCD279 (BD Biosciences) for 30 minutes at 4°C. Expression of CD274, CD273 and CD279 were analyzed using a flow cytometer (FACSCalibur or FACS LSR II, BD Biosciences).

For CD274 mRNA expression analysis, neutrophils were stimulated 0, 2, 4, 6 and 24 hours with IFN-γ, washed once with PBS and dissolved in RLT lysis buffer containing 1% β-mercaptoethanol and immediately frozen at −80°.

### Neutrophil survival

Neutrophils were suspended in HEPES-buffered RPMI supplemented with 10% FCS to a concentration of 5.10^6^/mL and stimulated with 100 ng/mL TNF-α (BD Biosciences), 50 ng/mL G-CSF (R&D systems), 50 ng/mL GM-CSF (Sanquin), 200 ng/mL IFN-α2 (Roche), 100 U/mL IFN-β1A (Invitrogen), 100 ng/mL IFN-γ (Sigma), 50 ng/mL LPS (Invitrogen) for 18–20 hours at 37°C+5% CO_2_. Apoptosis was determined by annexin-V binding (BD Biosciences). After staining the cells with annexin-V for 15 min in the dark at room temperature in annexin-binding buffer, 10 mM HEPES (pH 7.4), 140 mM NaCl, 2.5 mM CaCl_2_, 7-AAD were added. Cells were analyzed using a flow cytometer (FACSCalibur or FACS LSR II, BD Biosciences).

### Lymphocyte proliferation assay

Neutrophils were suspended in HEPES-buffered RPMI supplemented with 10% FCS to a concentration of 5.10^6^/mL and stimulated with either recombinant 50 ng/mL GM-CSF, 200 ng/mL IFN-α2 (Roche), 100 U/mL IFN-β1A (Invitrogen), 100 ng/mL IFN-γ (Sigma) or left untreated for 18–20 hours at 37°C+5% CO_2_. PBMCs from same donor were loaded with 5 μM CFSE (Sigma) and incubated 18–20 hours in HEPES-buffered RPMI supplemented with 10% pooled human AB-serum (Sigma) at 37°C+5% CO_2_. Neutrophils were washed twice with PBS, resuspended in HEPES-buffered RPMI supplemented with 10% pooled human AB-serum (Sigma) and added in various ratios to the CFSE-loaded PBMCs. Proliferation was stimulated with 5 μg/mL PHA (Sigma), CD3 (0.15 μg/mL)/CD28 (1 μg/mL) (Sanquin) or heat-killed *Candida albicans* (1.10^6^ CFU/mL) and measured by flow cytometry after 3 (PHA and CD3/CD28) or 7 days (*C. albicans*) with the gating strategy as described in supplemental figure S3. Blocking studies were performed with 10 μg/mL αCD274 (clone MIH1, eBiosciences), 10 μg/mL αCD11b (clone 44a, gift Prof. Leo Koenderman) or 10 μg/mL αPAFr (clone 11A4, Cayman Chemicals) as isotype control that were present throughout the 3 days incubation.

### Neutrophil-PBMC interaction

Neutrophils were suspended in HEPES-buffered RPMI supplemented with 10% FCS to a concentration of 5.10^6^/mL and stimulated with IFN-γ (Sigma) or left untreated for 18–20 hours at 37°C+5% CO_2_, washed once with PBS and loaded with 5 μM Calcein-Blue (Invitrogen) in PBS supplemented with 0.1% BSA after which the neutrophils were washed twice with PBS and suspended to a concentration of 4.10^6^/mL in RPMI supplemented with 2 mM L-Glutamine and 10% heat-inactivated human serum. PBMCs from the same donor were incubated 18–20 hours at 37°C+5% CO_2_, washed once with PBS and loaded with 1 μM Calcein-AM (Sigma) in PBS supplemented with 0.1% BSA after which the PBMCs were washed twice with PBS and suspended to a concentration of 2.10^6^/mL in RPMI supplemented with 2 mM L-Glutamine and 10% heat-inactivated human serum. Neutrophils and PBMC were mixed 2∶1, stimulated with 5 μg/mL PHA (Sigma) in the presence or absence of 10 μg/mL αCD11b (clone 44a) blocking antibody and Calcein-Blue and Calcein-AM double positive events were measured by flow cytometry (FACSCalibur or FACS LSR II, BD Biosciences).

### Statistics

Statistical analysis was performed by using Graphpad prism 5. Reported values are shown as mean with standard error of the mean (SEM). We used a t-test, one-way ANOVA with Tukey post hoc test or two-way ANOVA with Bonferoni multiple comparisons. P values of <0.05 were considered statistically significant.

## Results

### Neutrophil subsets mobilized during experimental human endotoxemia display distinctive transcriptome profiles

**Figure 1:**
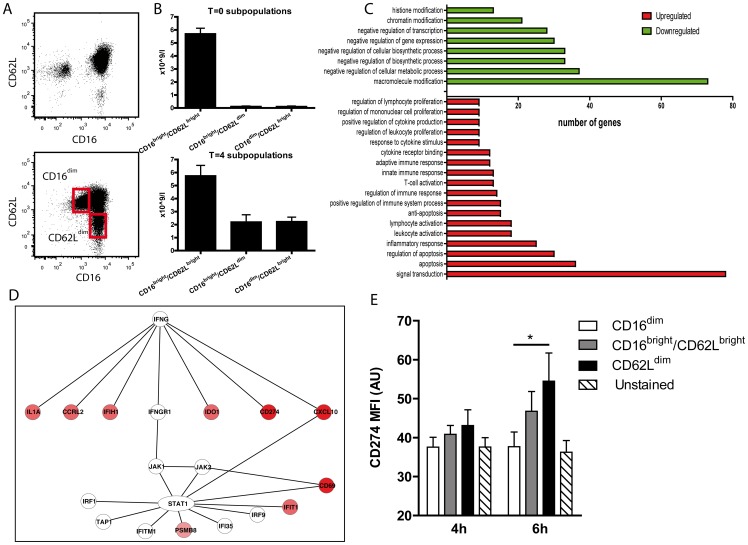
Microarray analysis of neutrophils subsets during human experimental endotoxemia. (**A**) Flow cytometry dot plot of neutrophils prior to, and 4 hours after *in vivo* LPS administration. The fluorescence signal for CD16 is displayed on the x-axis and the fluorescence signal for CD62L is displayed on the y-axis. At t = 0 hours (upper panel) a large population of CD16^bright^/CD62L^bright^ neutrophils, a small population of CD16^high^/CD62L^dim^ neutrophils and a population of CD16^negative^ cells representing eosinophils is present. At t = 4 hours after LPS (lower panel), neutrophil with CD16^dim^/CD62L^bright^ and CD16^dim^/CD62L^dim^ subsets appeared and these were FACS sorted for microarray analysis. (**B**) Absolute cell numbers of different neutrophil subsets in the blood at 0 and 4 hours after LPS challenge (n = 6). Data are expressed as means ± SEM. (**C**) Overrepresented functional categories in CD16^high^/CD62L^dim^ neutrophils based on the total list of differentially expressed genes relative to prior to LPS. A minimum of 5 genes and a p value of 0,01 were taken as cutoff. All significantly overrepresented categories are shown. The 4 highest parent levels of the Gene ontology tree were excluded for this graph since various general processes are involved in these. (**D**) Network of several interferon-induced genes that are upregulated in CD16^high^/CD62L^dim^ neutrophils after intravenous administration of LPS. The color intensity of the nodes indicates the level of upregulation. (**E**) Expression of CD274 on isolated neutrophil subsets from volunteers intravenous administered LPS at 4 and 6 hours after LPS. *P<0.05. Data are expressed as means ± SEM (n = 4).

Experimental human endotoxemia was used to induce systemic inflammation for mobilization of different neutrophil subsets into the circulation. Granulocytes were gated (Gating strategy described in supplemental figure S1) based on FSC/SSC, negative for CD14, and showed a large population of CD16^bright^/CD62L^bright^ neutrophils, a small population of CD16^bright^/CD62L^dim^ neutrophils and CD16^negative^/CD62L^high^ eosionophils (Figure 1A, upper panel). Four hours after a single intravenous dose of LPS (2 ng/kg body weight), 3 neutrophil subsets could be easily identified based on their expression of CD16 and CD62L (Figure 1A, lower panel). Hereafter, the different neutrophil phenotypes were isolated by FACS [5]. These subsets represented on average a CD16^bright^/CD62L^bright^ (62%), a CD16^dim^/CD62L^bright^ (19%) and a CD16^bright^/CD62L^dim^ (19%) phenotype (Figure 1B). Microarray analysis of these neutrophil subsets revealed a clear response to LPS administration with 819 (CD16^bright^/CD62L^bright^), 998 (CD16^bright^/CD62L^dim^) and 1108 (CD16^dim^/CD62L^bright^) genes differentially expressed at least 2-fold relative to neutrophils isolated prior to LPS administration (Table S1). A total number of 690 genes were persistently higher expressed throughout the neutrophil subsets with lowest expression in CD16^dim^/CD62L^bright^, intermediate expression in CD16^bright^/CD62L^bright^ and highest expression in CD16^bright^/CD62L^dim^ neutrophils. Gene ontology (GO)-term enrichment analysis of this set of genes showed overrepresentation of genes involved in regulation of immune responses and apoptosis, but also the regulation of leukocyte proliferation (Figure 1C). The top 50 genes increased in CD16^bright^/CD62L^dim^ neutrophils compared to normal CD16^bright^/CD62L^bright^ neutrophils were enriched for interferon signaling, which included increased expression of *CXCL10*, *IDO1*, *IL1A*, *CCRL2* and *CD274* (Figure 1D). Interestingly, from this list, expression of CD274 (the gene encoding for Programmed Death-Ligand 1, PD-L1), was highly increased in the suppressive CD16^bright^/CD62L^dim^ neutrophils compared to the CD16^bright^/CD62L^bright^ and CD16^dim^/CD62L^bright^ neutrophils. PD-L1 is a surface expressed ligand known to interact with its receptor PD-1 on various cell types to suppress cellular responses and proliferation [11]. We measured PD-L1 surface expression on the different neutrophil subsets 4 and 6 hours post-endotoxin administration. The surface protein expression of PD-L1 was significantly higher on CD16^bright^/CD62L^dim^ neutrophils compared to CD16^dim^/CD62L^bright^ neutrophils, and intermediate on CD16^bright^/CD62L^bright^ neutrophils (Figure 1E).

### Ex vivo stimulation of neutrophils with IFN-γ induces expression of PD-L1

**Figure 2:**
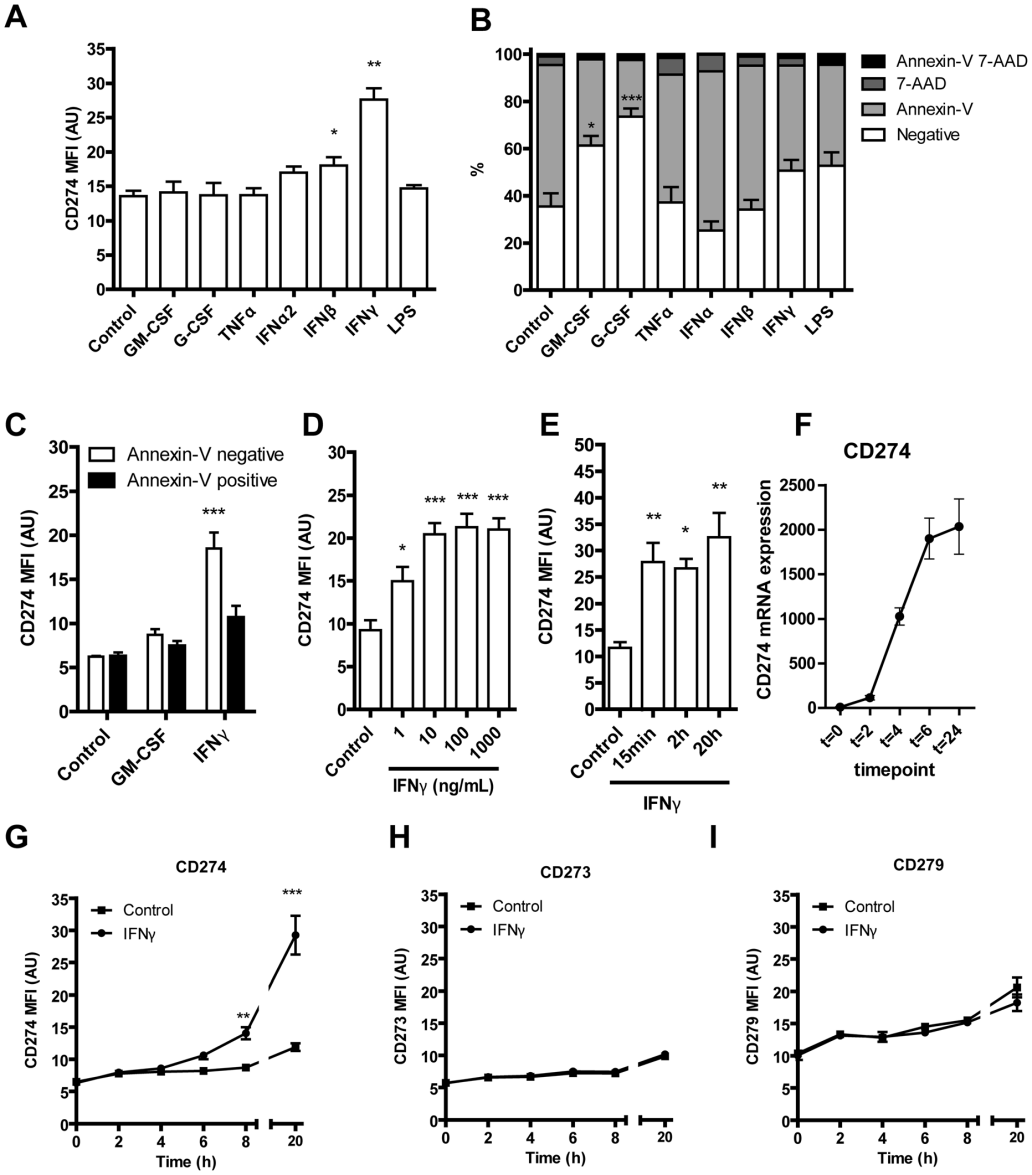
PD-L1 expression on IFN-γ treated neutrophils. (**A**) Neutrophils were stimulated 18–20 hours with different cytokines and growth factors and CD274 mean fluorescence intensity (MFI) was measured. (**B**) Neutrophil survival after 18–20 hours stimulation with different cytokines and growth factors shown on the x-axis and the percentage of cells that were positive for either Annexin-V, 7-AAD or both on the y-axis. (**C**) Freshly isolated neutrophils were stimulated 18–20 hours with IFN-γ or GM-CSF and CD274 mean fluorescence intensity (MFI) was measured on annexin-V negative and annexin-V positive neutrophils (**D**) Neutrophils were stimulated 18–20 hours with different concentrations of IFN-γ and CD274 mean fluorescence intensity (MFI) was measured. (**E**) Neutrophils were stimulated with 100 ng/ml IFN-γ and incubated for different periods before washing and further incubation till 18–20 hours and CD274 mean fluorescence intensity (MFI) was measured. (**F**) Gene expression of CD274 in IFN-γ stimulated neutrophils. Expression is shown in time with the use of GAPDH as reference gene. Surface expression of (**G**) CD274, (**H**) CD273 and (**I**) CD279 after 0, 2, 4, 6, 8 and 20 hours stimulation with IFN-γ. *P<0.05, **P<0.01, ***P<0.001. Data are expressed as means ± SEM (n = 4).

With regard to the pronounced IFN-induced profile in CD16^bright^/CD62L^dim^ neutrophils, we further investigated the role of IFN signaling in the generation of suppressive neutrophils by stimulating freshly isolated neutrophils with various cytokines or LPS. Interestingly, especially IFN-γ, and to a lesser extend IFN-α or IFN-β, but not G-CSF, GM-CSF, TNF-α, LPS, increased PD-L1 expression as measured by flow cytometry (Figure 2A, and representative histograms in supplemental figure S2). Expression of PD-L1 could not be directly related to increased survival of the neutrophils by IFN-γ, since GM-CSF and G-CSF did not increase expression of PD-L1, but did increase survival (Figure 2B).

Subsequently, we assessed the dynamics of PD-L1 expression on IFN-γ-stimulated neutrophils. Expression of PD-L1 was especially apparent on annexin-V negative neutrophils (Figure 2C). Treatment with 1 ng/ml IFN-γ was sufficient to induce PD-L1 expression, reaching a plateau at 10–100 ng/ml (Figure 2D). Stimulation of neutrophils with IFN-γ for only 15 minutes was sufficient to induce PD-L1 expression after 18–20 hours (Figure 2E). Stimulation of neutrophils with IFN-γ increased CD274 mRNA expression starting at 2 hours, and reached maximum after 6 hours (Figure 2F). Subsequently, we determined expression of PD-L1 on neutrophils in time. Stimulation of neutrophils with IFN-γ-induced PD-L1 surface expression slightly after 6 hours, which increased after 8 and 20 hours (Figure 2G), whereas this was not detected for CD273 (PD-L2) (Figure 2H) or CD279 (PD-1) (Figure 2I).

### IFN-γ-stimulated neutrophils suppress lymphocyte proliferation

**Figure 3:**
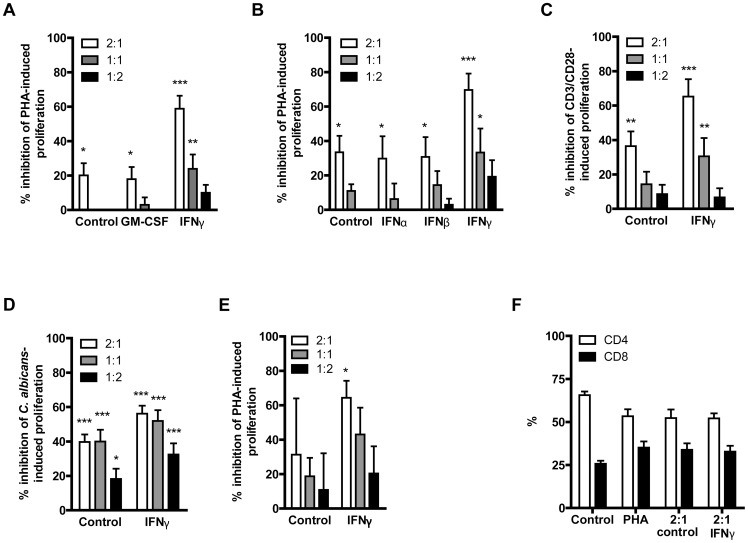
IFN-γ stimulated neutrophils suppress T-cell proliferation. (**A**) Neutrophils stimulated 18–20 hours with either GM-CSF or IFN-γ or left untreated and inhibition of PHA-induced PBMC proliferation was measured after 3 days. (**B**) Neutrophils stimulated 18–20 hours with IFN-α, IFN-β, IFN-γ or left untreated and inhibition of PHA-induced PBMC proliferation was measured after 3 days. (**C**) Neutrophils stimulated 18–20 hours with IFN-γ or left untreated and inhibition of CD3/CD28-induced PBMC proliferation was measured after 3 days. (**D**) Neutrophils stimulated 18–20 hours with IFN-γ or left untreated and inhibition of *Candida albicans*-induced proliferation was measured after 7 days. (**E**) CD16^positive^ CD14^negative^ CD3^negative^ sorted neutrophils were stimulated 18–20 hours with IFN-γ or left untreated and inhibition of PHA-induced CD3^positive^ CD14^negative^ CD16^negative^ sorted lymphocyte proliferation was measured after 3 days. (**F**) Percentage of CD4 and CD8 lymphocytes after 3 days PHA-induced PBMC proliferation in the presence of neutrophils stimulated 18–20 hours with IFN-γ or left untreated.

We determined the capacity of neutrophils that were stimulated with different types of interferons or GM-CSF to suppress lymphocyte proliferation (Gating strategy described in supplemental figure S3). Untreated, GM-CSF-, IFN-α- and IFN-β-stimulated neutrophils showed modest suppression of phytohemagglutinin (PHA)-induced lymphocyte proliferation, whereas IFN-γ-stimulated neutrophils showed a robust, up to 70%, inhibition of proliferation (Figure 3A–B and supplemental figure S3). Suppression of proliferation was also observed when lymphocytes were activated by CD3/CD28 (Figure 3C) or *Candida albicans* (Figure 3D), which points toward a general mechanism of suppression. To exclude a role for other leukocytes such as monocytes or eosinophils in the suppression of lymphocyte proliferation, neutrophils (CD16^positive^, CD14^negative^, CD3^negative^) and lymphocytes (CD16^negative^, CD14^negative^, CD3^positive^) were sorted by FACS. IFN-γ-stimulated purified neutrophils showed increased capacity to suppress PHA-induced purified lymphocyte proliferation compared to untreated neutrophils (Figure 3E). Although the induction of PHA-induced proliferation was decreased (data not shown), the level of suppression was comparable with total cell populations, indicating that this process is not dependent on the presence of other cell types such as monocytes.

Subsequently, lymphocyte phenotype after co-culture with neutrophils was analyzed. There was no significant difference in the distribution of CD4 and CD8 lymphocytes after co-stimulation with either IFN-γ-stimulated or control neutrophils (Figure 3F).

### IFN-γ-induced PBMC suppression is dependent on cell-cell contact and PD-L1

**Figure 4:**
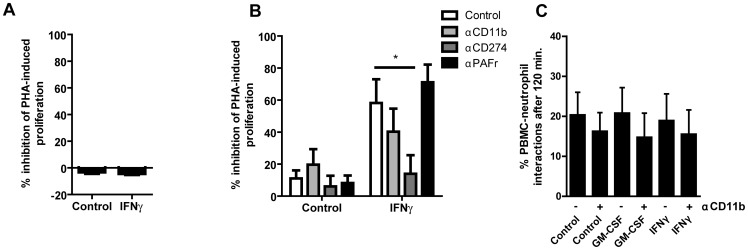
Suppression of T-cell proliferation by IFN-γ stimulated neutrophils is dependent on cell-cell contact and PD-L1. (**A**) Neutrophils stimulated with IFN-γ or left untreated for 18–20 hours and PHA-stimulated PBMCs were co-cultured for 3 days in separate compartments by the use of cell culture inserts. Percentage of inhibition of PHA-induced proliferation was calculated. (**B**) Neutrophils stimulated 18–20 hours with IFN-γ or left untreated and inhibition of PHA-induced PBMC proliferation in the presence of αCD11b, αCD274 or αPAFr was measured after 3 days. (**C**) Neutrophils stimulated 18–20 hours with either GM-CSF or IFN-γ or left untreated and interactions between Calcein-blue labeled neutrophils with Calcein-AM labeled PBMCs after 120 minutes of co-culture measured by flow cytometer. Ratios indicate neutrophils: lymphocytes (Figure A–E). *P<0.05, **P<0.01, ***P<0.001. Data are expressed as means ± SEM (n = 4).

Next, we investigated whether neutrophil-mediated T-cell suppression was dependent on cell-cell contact between lymphocytes and IFN-γ-stimulated neutrophils using a transwell system separating both cell suspensions. In this system, neutrophils lost their suppressive capacity indicating that cellular proximity is needed between neutrophils and lymphocytes (Figure 4A).

Previously it was postulated that CD16^bright^/CD62L^dim^ neutrophils form a synapse with lymphocytes wherein integrin MAC-1 (CD11b) plays a pivotal role [6]. We hypothesized that, next to the formation of a synapse by integrin MAC-1 on neutrophils, expression of PD-L1 would contribute to the suppressive function on lymphocyte proliferation because expression of this molecule on other cell types enables suppression of lymphocyte activation and proliferation [11]. Blocking MAC-1 using monoclonal antibody 44a in our co-cultures showed a modest decrease in suppressive capacity (Figure 4B). As hypothesized, PD-L1 showed to be absolutely essential for the IFN-γ-induced suppressive effect, because blocking PD-L1 attenuated suppression to the level of unstimulated neutrophils, whereas the isotype control antibody showed no effect (Figure 4B). In order to evaluate the role of MAC-1 and IFN-γ on the interaction between neutrophils and lymphocytes, we loaded neutrophils with Calcein-Blue and PBMCs with Calcein-AM. Interactions between these cell types were visualized as double positive events by flow cytometry. The percentage double positive events increased to more than 20% in 120 minutes of co-culture, which decreased to 15% in the presence of αCD11b (Figure 4C). However, no differences were found between IFN-γ-, GM-CSF-stimulated or unstimulated neutrophils. Therefore, we conclude that interactions between neutrophils and lymphocytes occur independently of stimulation, but only in the presence of PD-L1 expression neutrophils inhibit lymphocyte proliferation.

## Discussion

In the present study, we show that the neutrophil subsets that appear in the circulation during systemic inflammation elicited by experimental human endotoxemia, have distinct gene expression profiles. Our gene expression data indicate that for a significant amount of genes, the expression increases on a gradual scale with lowest expression in CD16^dim^/CD62L^bright^ neutrophils, intermediate expression in CD16^bright^/CD62L^bright^ neutrophils and the highest expression in CD16^bright^/CD62L^dim^ neutrophils. With regard to their pronounced inflammatory gene expression pattern and hypersegmented nucleus, it can be suggested that CD16^bright^/CD62L^dim^ neutrophils are representative of a later phase in the lifespan of neutrophils. The origin of these CD16^bright^/CD62L^dim^ neutrophils is currently unknown. Since CD62L is shed from activated neutrophils, this marker has limitations in defining a homogenous subset of neutrophils [12]. The CD16^bright^/CD62L^dim^ neutrophil subset, obtained during experimental human endotoxemia, is clearly able to suppress lymphocyte proliferation in contrast to the CD16^dim^/CD62L^dim^ neutrophils [6].

We sought to investigate the factors involved in the generation of CD16^bright^/CD62L^dim^ suppressive neutrophil subset during systemic inflammation *in vivo* based on their transcriptome. Our gene expression data showed upregulation of various IFN-induced genes during endotoxemia, which was most pronounced in the CD16^bright^/CD62L^dim^ neutrophil subset. Previously, it has been shown that stimulation of whole blood with IFN-γ + GM-CSF induces expression of IFN-regulated genes *CXCL10*, *IDO1*, *IL1A*, *CCRL2* and *CD274* [13], a profile that resembles the transcriptome of CD16^bright^/CD62L^dim^ neutrophils.

The moment of increased surface expression of PD-L1 on neutrophils during experimental human endotoxemia (6 hours post LPS), compared to our *ex vivo* experiments (6–8 hours post IFN-γ), which suggests that neutrophils are exposed to IFN-γ shortly after LPS infusion. The main producers of IFN-γ are CD4+ Th1 lymphocytes, CD8+ cytotoxic lymphocytes and natural killer (NK) cells [14]. These cell types typically do not respond directly to LPS, therefore, an indirect effect of LPS on the release of IFN-γ appears more likely. For instance, it was recently shown that flagellin-induced rapid IL-18 release from dendritic cells, which induced IFN-γ release from memory CD8+ T cells within 2 hours [15]. However, whether a similar indirect mechanism is responsible for IFN-γ release after LPS administration remains to be determined. Currently, we have no evidence that IFN-γ induces PD-L1 expression on neutrophils *in vivo*, or whether PD-L1 on CD16^bright^/CD62L^dim^ neutrophils is essential for the suppressive capacity observed by Pillay and co-workers [6]. The data we present here do support an important role for PD-L1 on neutrophils in lymphocyte proliferation *in vitro*.

The induction of PD-L1 on IFN-γ-stimulated neutrophils is likely de novo synthesis since a recent study did not detect this protein in the granules [16]. Expression of PD-L1 on circulating neutrophils has been shown in patients with active tuberculosis [17]. This is especially interesting because neutrophils from patients with active tuberculosis also exhibit an IFN-induced transcriptome profile, including increased expression of *CXCL10* and *CD274* [18]. To date, no studies have investigated PD-L1 expression on neutrophils during systemic inflammatory diseases. Interestingly, during sepsis, increased expression of PD-L1 on monocytes has been suggested to play an important role in sepsis-induced immunosuppression [19], [20].

To our knowledge, we are the first to identify an immune-suppressive effect of IFN-γ through expression of PD-L1 on neutrophils. Although originally defined as an agent with direct antiviral activity, the properties of IFN-γ also include regulation of several neutrophil functions such as stimulation of the respiratory burst [21], increased *ex vivo* survival [22] and antigen presentation [23]. We demonstrate that the IFN-γ-induced suppression of lymphocyte proliferation is dependent on increased expression of PD-L1. Under steady state conditions, expression of PD-L1 on neutrophils is very low [23] and these neutrophils show only a minor suppressive phenotype *ex vivo*. The suppressive phenotype on lymphocyte proliferation was independent of the stimulation method, as similar findings were observed for PHA, CD3/CD28 and *Candida albicans* stimulation. The suppressive capacity of the IFN-γ-stimulated neutrophils, as shown by neutrophil lymphocyte co-culture in transwell experiments, occurred in a cell-cell contact dependent manner. IFN-γ has been shown to increase expression of PD-L1 on various cell types [24] resulting in suppressive activity through ligation with PD-1 on target cells [25]. By blocking PD-L1 on neutrophils we verified that suppression of lymphocyte proliferation was dependent on PD-L1 – PD1 signaling.

This study shows for the first time that suppressive neutrophils can be generated using IFN-γ, which could be used as a novel approach to modulate inflammation. For instance, during influenza infections, the tissue damage that is associated with disease pathology is dependent on the presence of T-cells [26]. In this case, more damage is caused by the host’s inflammatory response compared with damage caused by the virus itself. In order to maintain balance in inflammatory responses and to prevent excessive tissue damage, other immune cells such as dendritic cells and macrophages, but as suggested by our data also neutrophils, dampen excessive T-cell responses. This hypothesis is supported by the fact that neutrophil depletion in influenza-infected mice leads to aggravated disease characterized by rapid weight loss, pneumoniae and death [27], [28].

In conclusion, stimulation of peripheral blood neutrophils with IFN-γ *ex vivo* induces PD-L1 expression on neutrophils, which is shown to be essential in the suppression of lymphocyte proliferation *in vitro*. Therefore, IFN-γ-stimulated neutrophils might provide a novel therapeutic option for the reduction of T-cell mediated tissue damage in inflammatory diseases.

## Supporting Information

Figure S1 **FACS gating strategy of sorted neutrophil subsets.** Whole blood was shocked and labeled with antibodies. First, granulocytes were gated based on forward/sideward scatter (upper panels). Then CD14- granulocytes were selected (mid panels). Then neutrophil subsets were selected based on CD16 and CD62L expression (lower panels).
(PDF)

Figure S2 **Neutrophil CD274 expression gating strategy.** (A) Neutrophils were selected on the basis of their FSC/SSC. (B) MFI of the whole granulocyte population was determined. (C) Overlay of PD-L1 expression of unstimulated, IFNα, IFNβand IFNγ-stimulated neutrophils.
(PDF)

Figure S3 **Lymphocyte proliferation assay gating strategy.** (A) Lymphocytes were selected on the basis of their FSC/SSC. (B) FITC-positive events were selected based on the PHA-stimulated lymphocytes to exclude inclusion of apoptotic neutrophils. (C) The gate % proliferation was selected based on the unstimulated lymphocytes. All gates were identical in all samples within one experiment.
(PDF)

Table S1 Microarray analysis of neutrophil subsets. Genes differentially expressed at least 2-fold relative to neutrophils isolated prior to LPS administration are depicted.
(PDF)

## References

[1] Borregaard N (2010) Neutrophils, from marrow to microbes. Immunity 33: 657–670. 21094463 10.1016/j.immuni.2010.11.011

[2] Mantovani A, Cassatella MA, Costantini C, Jaillon S (2011) Neutrophils in the activation and regulation of innate and adaptive immunity. Nat Rev Immunol 11: 519–531. 21785456 10.1038/nri3024

[3] van Gisbergen KP, Ludwig IS, Geijtenbeek TB, van Kooyk Y (2005) Interactions of DC-SIGN with Mac-1 and CEACAM1 regulate contact between dendritic cells and neutrophils. FEBS Lett 579: 6159–6168. 16246332 10.1016/j.febslet.2005.09.089

[4] Megiovanni AM, Sanchez F, Robledo-Sarmiento M, Morel C, Gluckman JC, et al (2006) Polymorphonuclear neutrophils deliver activation signals and antigenic molecules to dendritic cells: a new link between leukocytes upstream of T lymphocytes. J Leukoc Biol 79: 977–988. 16501052 10.1189/jlb.0905526

[5] Pillay J, Ramakers BP, Kamp VM, Loi AL, Lam SW, et al (2010) Functional heterogeneity and differential priming of circulating neutrophils in human experimental endotoxemia. JLeukocBiol 88: 211–220. 10.1189/jlb.1209793 20400675

[6] Pillay J, Kamp VM, van Hoffen E, Visser T, Tak T, et al (2012) A subset of neutrophils in human systemic inflammation inhibits T cell responses through Mac-1. J Clin Invest 122: 327–336. 22156198 10.1172/JCI57990 PMC3248287

[7] de Kleijn S, Kox M, Sama IE, Pillay J, van Diepen A, et al (2012) Transcriptome kinetics of circulating neutrophils during human experimental endotoxemia. PLoS One 7: e38255. 22679495 10.1371/journal.pone.0038255 PMC3367952

[8] Leentjens J, Kox M, Koch RM, Preijers F, Joosten LA, et al (2012) Reversal of Immunoparalysis in Humans In Vivo: A Double-Blind, Placebo-controlled, Randomized Pilot Study. Am J Respir Crit Care Med 186: 838–845. 22822024 10.1164/rccm.201204-0645OC

[9] Gellert P, Uchida S, Braun T (2009) Exon Array Analyzer: a web interface for Affymetrix exon array analysis. Bioinformatics 25: 3323–3324. 19808879 10.1093/bioinformatics/btp577

[10] Langereis JD, Franciosi L, Ulfman LH, Koenderman L (2011) GM-CSF and TNFalpha modulate protein expression of human neutrophils visualized by fluorescence two-dimensional difference gel electrophoresis. Cytokine 56: 422–429. 21873076 10.1016/j.cyto.2011.06.025

[11] Keir ME, Butte MJ, Freeman GJ, Sharpe AH (2008) PD-1 and its ligands in tolerance and immunity. Annu Rev Immunol 26: 677–704. 18173375 10.1146/annurev.immunol.26.021607.090331 PMC10637733

[12] Hayashi F, Means TK, Luster AD (2003) Toll-like receptors stimulate human neutrophil function. Blood 102: 2660–2669. 12829592 10.1182/blood-2003-04-1078

[13] Kotz KT, Xiao W, Miller-Graziano C, Qian WJ, Russom A, et al (2010) Clinical microfluidics for neutrophil genomics and proteomics. NatMed 16: 1042–1047. 10.1038/nm.2205 PMC3136804 20802500

[14] Boehm U, Klamp T, Groot M, Howard JC (1997) Cellular responses to interferon-gamma. Annu Rev Immunol 15: 749–795. 9143706 10.1146/annurev.immunol.15.1.749

[15] Kupz A, Guarda G, Gebhardt T, Sander LE, Short KR, et al (2012) NLRC4 inflammasomes in dendritic cells regulate noncognate effector function by memory CD8(+) T cells. Nat Immunol 13: 162–169. 22231517 10.1038/ni.2195

[16] Rorvig S, Ostergaard O, Heegaard NH, Borregaard N (2013) Proteome profiling of human neutrophil granule subsets, secretory vesicles, and cell membrane: correlation with transcriptome profiling of neutrophil precursors. J Leukoc Biol. 10.1189/jlb.1212619 23650620

[17] McNab FW, Berry MP, Graham CM, Bloch SA, Oni T, et al (2011) Programmed death ligand 1 is over-expressed by neutrophils in the blood of patients with active tuberculosis. Eur J Immunol 41: 1941–1947. 21509782 10.1002/eji.201141421 PMC3179592

[18] Berry MP, Graham CM, McNab FW, Xu Z, Bloch SA, et al (2010) An interferon-inducible neutrophil-driven blood transcriptional signature in human tuberculosis. Nature 466: 973–977. 20725040 10.1038/nature09247 PMC3492754

[19] Zhang Y, Li J, Lou J, Zhou Y, Bo L, et al (2011) Upregulation of programmed death-1 on T cells and programmed death ligand-1 on monocytes in septic shock patients. Crit Care 15: R70. 21349174 10.1186/cc10059 PMC3222003

[20] Hotchkiss RS, Opal S (2010) Immunotherapy for sepsis--a new approach against an ancient foe. NEnglJMed 363: 87–89. 10.1056/NEJMcibr1004371 PMC4136660 20592301

[21] Cassatella MA, Bazzoni F, Flynn RM, Dusi S, Trinchieri G, et al (1990) Molecular basis of interferon-gamma and lipopolysaccharide enhancement of phagocyte respiratory burst capability. Studies on the gene expression of several NADPH oxidase components. J Biol Chem 265: 20241–20246. 2173701

[22] Yoshimura T, Takahashi M (2007) IFN-gamma-mediated survival enables human neutrophils to produce MCP-1/CCL2 in response to activation by TLR ligands. J Immunol 179: 1942–1949. 17641061 10.4049/jimmunol.179.3.1942

[23] Bankey PE, Banerjee S, Zucchiatti A, De M, Sleem RW, et al (2010) Cytokine induced expression of programmed death ligands in human neutrophils. Immunol Lett 129: 100–107. 20123111 10.1016/j.imlet.2010.01.006 PMC2849642

[24] Sheng H, Wang Y, Jin Y, Zhang Q, Zhang Y, et al (2008) A critical role of IFNgamma in priming MSC-mediated suppression of T cell proliferation through up-regulation of B7-H1. Cell Res 18: 846–857. 18607390 10.1038/cr.2008.80

[25] Freeman GJ, Long AJ, Iwai Y, Bourque K, Chernova T, et al (2000) Engagement of the PD-1 immunoinhibitory receptor by a novel B7 family member leads to negative regulation of lymphocyte activation. J Exp Med 192: 1027–1034. 11015443 10.1084/jem.192.7.1027 PMC2193311

[26] Rygiel TP, Rijkers ES, de Ruiter T, Stolte EH, van der Valk M, et al (2009) Lack of CD200 enhances pathological T cell responses during influenza infection. J Immunol 183: 1990–1996. 19587022 10.4049/jimmunol.0900252

[27] Tate MD, Deng YM, Jones JE, Anderson GP, Brooks AG, et al (2009) Neutrophils ameliorate lung injury and the development of severe disease during influenza infection. J Immunol 183: 7441–7450. 19917678 10.4049/jimmunol.0902497

[28] Fujisawa H (2008) Neutrophils play an essential role in cooperation with antibody in both protection against and recovery from pulmonary infection with influenza virus in mice. J Virol 82: 2772–2783. 18184718 10.1128/JVI.01210-07 PMC2258992

