# Supplementary material for: IFN-γ-Stimulated Neutrophils Suppress Lymphocyte Proliferation through Expression of PD-L1
Source: PLoS One. 2013 Aug 28;8(8):e72249. doi: 10.1371/journal.pone.0072249 (PMC3756078; doi:10.1371/journal.pone.0072249)
Supplement: Figure S1 — FACS gating strategy of sorted neutrophil subsets. Whole blood was shocked and labeled with antibodies. First, granulocytes were gated based on forward/sideward scatter (upper panels). Then CD14- granulocytes were selected (mid panels). Then neutrophil subsets were selected based on CD16 and CD62L expression (lower panels). (PDF) [file pone.0072249.s001.pdf]

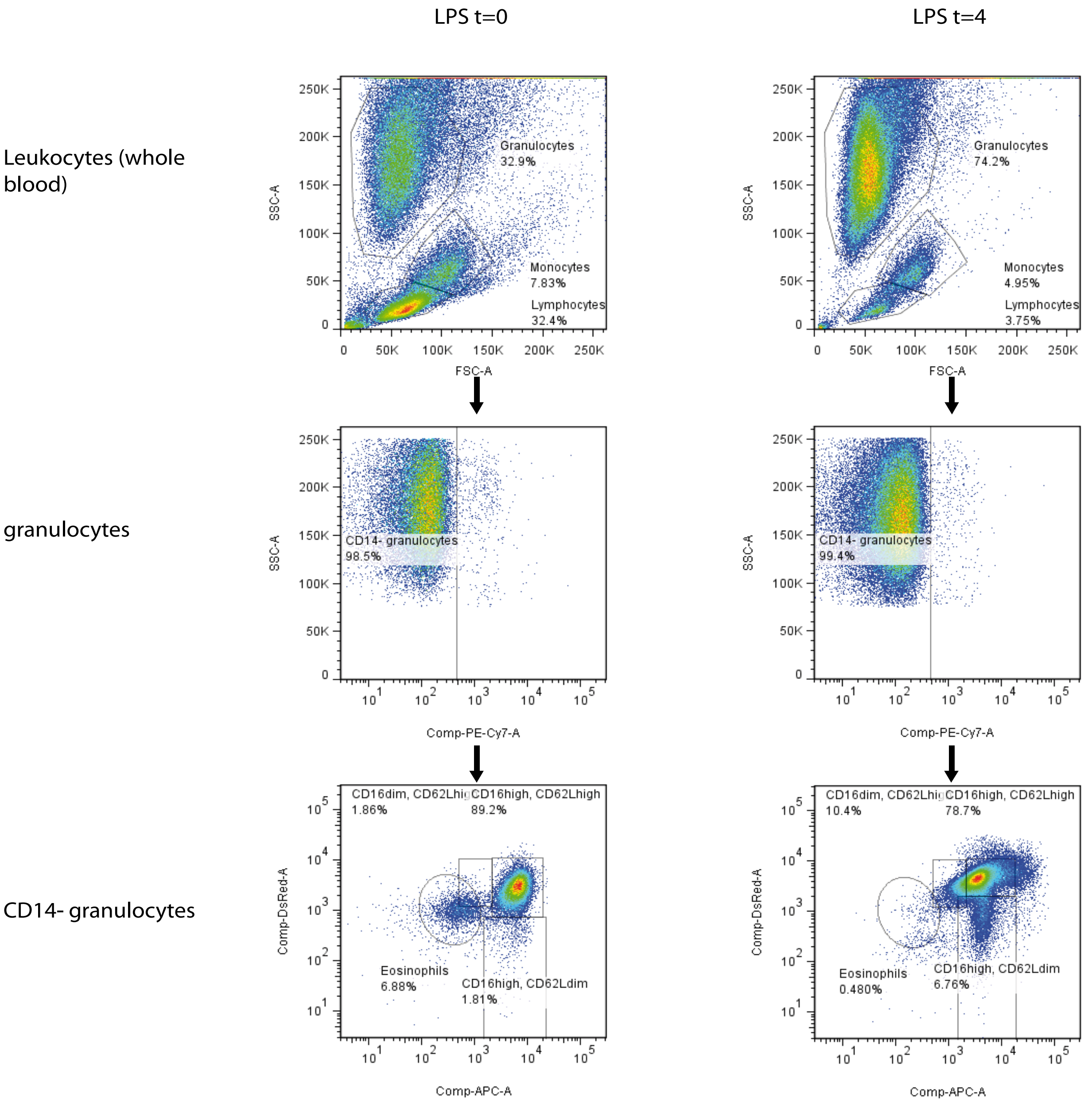

Supplemental figure S1. FACS gating strategy of sorted neutrophil subsets. Whole blood was shocked and labeled with antibodies. First, granulocytes were gated based on forward/sideward scatter (upper panels). Then CD14- granulocytes were selected (mid panels). Then neutrophil subsets were selected based on CD16 and CD62L expression (lower panels)
