# Supplementary material for: IFN-γ-Stimulated Neutrophils Suppress Lymphocyte Proliferation through Expression of PD-L1
Source: PLoS One. 2013 Aug 28;8(8):e72249. doi: 10.1371/journal.pone.0072249 (PMC3756078; doi:10.1371/journal.pone.0072249)
Supplement: Figure S2 — Neutrophil CD274 expression gating strategy. (A) Neutrophils were selected on the basis of their FSC/SSC. (B) MFI of the whole granulocyte population was determined. (C) Overlay of PD-L1 expression of unstimulated, IFNα, IFNβand IFNγ-stimulated neutrophils. (PDF) [file pone.0072249.s002.pdf]

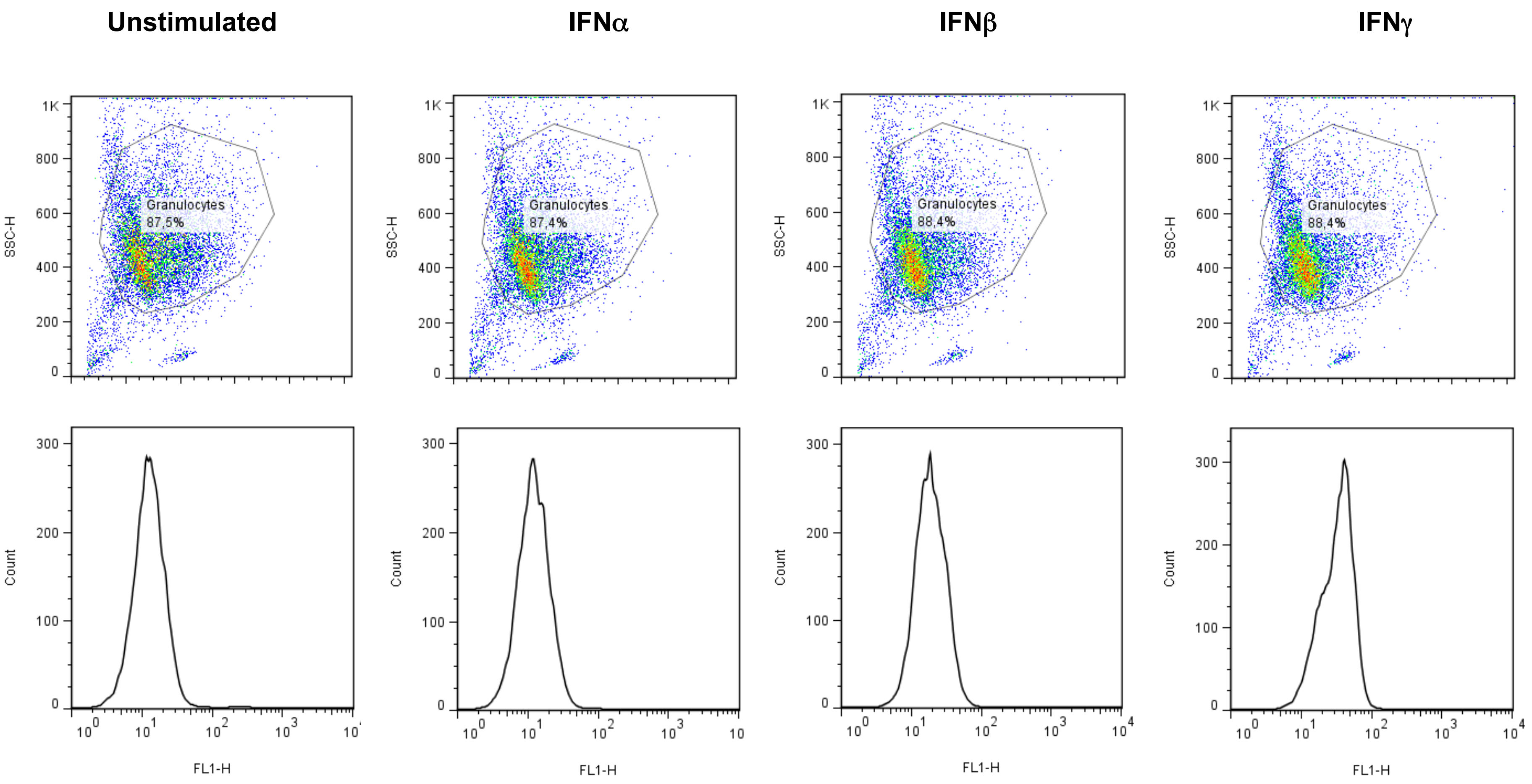

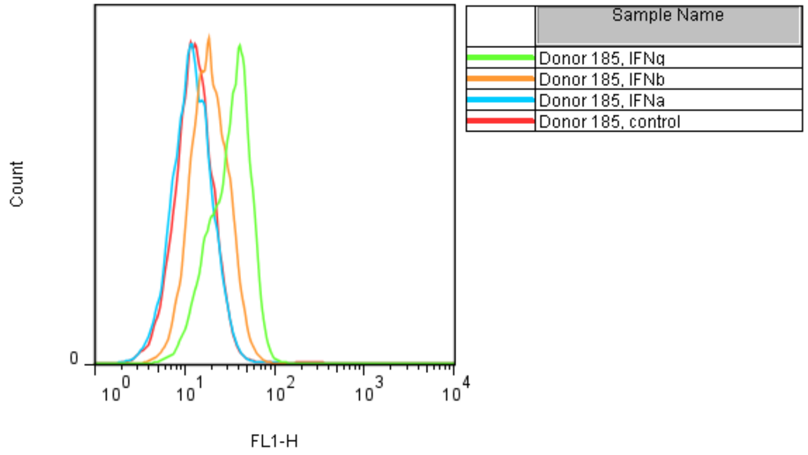

Supplemental figure S2. Neutrophil CD274 expression gating strategy. (A) Neutrophils were selected on the basis of their FSC/SSC. **(B)** MFI of the whole granulocyte population was determined. **(C)** Overlay of PD-L1 expression of unstimulated, IFNα, IFNβ and IFNγ-stimulated neutrophils
