# Supplementary material for: IFN-γ-Stimulated Neutrophils Suppress Lymphocyte Proliferation through Expression of PD-L1
Source: PLoS One. 2013 Aug 28;8(8):e72249. doi: 10.1371/journal.pone.0072249 (PMC3756078; doi:10.1371/journal.pone.0072249)
Supplement: Figure S3 — Lymphocyte proliferation assay gating strategy. (A) Lymphocytes were selected on the basis of their FSC/SSC. (B) FITC-positive events were selected based on the PHA-stimulated lymphocytes to exclude inclusion of apoptotic neutrophils. (C) The gate % proliferation was selected based on the unstimulated lymphocytes. All gates were identical in all samples within one experiment. (PDF) [file pone.0072249.s003.pdf]

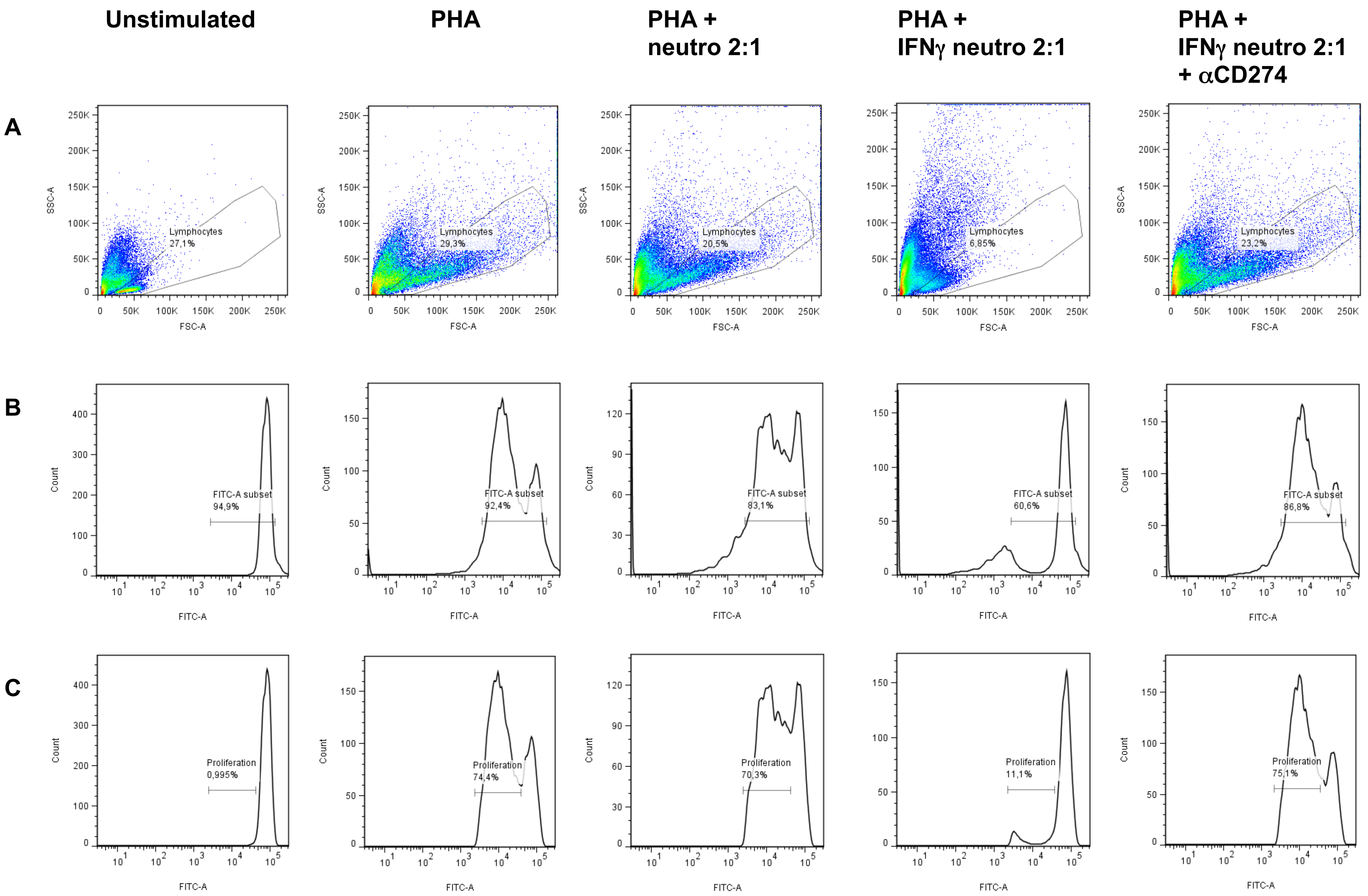


**Supplemental figure S3. Lymphocyte proliferation assay gating strategy. (A)** Lymphocytes were selected on the basis of their FSC/SSC. **(B)** FITC-positive events were selected based on the PHA-stimulated lymphocytes to exclude inclusion of apoptotic neutrophils. **(C)** The gate % proliferation was selected based on the unstimulated lymphocytes. All gates were identical in all samples within one experiment.
