# Supplementary material for: IFN-γ-Stimulated Neutrophils Suppress Lymphocyte Proliferation through Expression of PD-L1
Source: PLoS One. 2013 Aug 28;8(8):e72249. doi: 10.1371/journal.pone.0072249 (PMC3756078; doi:10.1371/journal.pone.0072249)
Supplement: Table S1 — Microarray analysis of neutrophil subsets. Genes differentially expressed at least 2-fold relative to neutrophils isolated prior to LPS administration are depicted. (PDF) [file pone.0072249.s004.pdf]

A= CD16bright/CD62Lbright at t=0
B= CD16bright/CD62Lbright at t=4
C= CD16bright/CD62Ldim at t=4
D= CD16dim/CD62Lbright at t=4

	Fold change			
gene name	BvsA_	CvsA_	DvsA_	CvsD_
37012	6.52	4.22	7.41	-1.75
39875	16.78	15.28	10.86	1.41
39879	-1.08	-1.25	-2.33	1.87
39880	-2.03	-2.65	-2.77	1.05
40058	1.00	-2.82	-1.60	-1.77
40063	-1.04	-1.65	-3.05	1.85
ABCA1	1.21	1.11	-1.88	2.10
ABCF1	1.44	2.12	1.24	1.71
ABHD2	-1.85	-2.46	-2.01	-1.22
ABHD3	-1.66	-1.95	-2.90	1.49
ABHD5	-1.10	-1.15	-2.19	1.91
ABTB1	-1.62	-1.83	-2.17	1.18
ACAP1	1.23	-1.45	1.67	-2.42
ACAP2	-1.30	-1.62	-2.07	1.28
ACBD5	-2.30	-2.08	-2.34	1.12
ACER3	2.68	1.19	2.41	-2.02
ACLY	-1.70	-2.21	-1.55	-1.43
ACN9	2.10	1.04	2.07	-1.99
ACOT13	-2.30	-1.75	-3.38	1.94
ACPP	-2.59	-3.50	-2.51	-1.40
ACSL3	3.41	1.75	1.75	-1.00
ACSL4	2.27	1.30	1.17	1.12
ACSL5	3.46	4.62	1.64	2.82
ACVR1B	2.18	1.44	2.04	-1.42
ADAM10	-1.87	-1.86	-2.74	1.47
ADAM17	1.22	1.48	-1.95	2.89
ADAT1	2.04	1.83	1.77	1.03
ADI1	-1.62	-2.00	-1.79	-1.12
ADK	-1.73	-1.69	-2.37	1.41
ADM	2.01	2.40	1.40	1.72
ADORA2A	1.74	3.00	1.75	1.71
ADRBK2	1.48	2.55	-1.38	3.51
ADSS	-2.05	-2.11	-2.15	1.02
AGFG1	2.01	1.32	2.18	-1.65
AGL	1.20	-1.29	1.66	-2.15
AGPAT6	3.12	2.40	2.18	1.10
AHSA2	-1.44	-1.69	-2.07	1.23
AIF1	-2.18	-1.39	-3.47	2.50
AIM2	1.57	1.39	-1.99	2.76
AK2	1.41	1.68	-1.46	2.46
AKAP8L	-2.12	-2.27	-1.86	-1.22
AKT1	-2.28	-2.70	-2.67	-1.01
AKTIP	-1.87	-3.19	-1.24	-2.58

ALCAM	-1.51	1.24	-2.66	3.29
ALDH2	3.83	2.57	2.05	1.26
ALDH9A1	-1.73	-1.69	-2.62	1.55
ALKBH5	-2.06	-2.06	-1.68	-1.22
AMACR	-1.87	-2.08	-1.88	-1.11
AMBRA1	-1.69	-1.87	-2.08	1.11
AMD1	-2.73	-2.78	-3.94	1.42
AMPD2	-4.95	-3.42	-5.11	1.50
AMY2B	-1.29	-1.71	-2.49	1.46
AMZ2	-2.05	-2.50	-2.06	-1.21
ANAPC13	-6.86	-4.13	-10.50	2.54
ANKRD12	1.93	1.89	-1.16	2.19
ANKRD22	11.16	8.69	7.34	1.19
ANKRD32	-1.70	-2.17	-2.04	-1.06
ANKRD36B	1.71	1.55	2.79	-1.81
ANKRD44	-1.30	-1.67	-2.19	1.31
ANKRD55	2.34	1.41	2.13	-1.51
ANXA1	1.51	-1.14	2.42	-2.77
ANXA3	3.94	2.80	2.79	1.01
ANXA7	1.42	1.86	-1.37	2.56
AOC2	-2.29	-2.10	-2.41	1.15
AOC3	-2.69	-2.25	-2.63	1.17
AP1M1	-1.73	-2.60	-1.20	-2.17
AP2M1	-1.62	-2.09	-1.63	-1.28
AP3B1	-1.32	-1.79	-2.55	1.42
AP3M1	-2.47	-2.81	-2.02	-1.39
APAF1	-1.32	-2.08	-1.40	-1.49
APC	-1.52	-1.66	-2.34	1.41
APOBEC3B	-1.32	1.36	-2.09	2.84
APOL1	1.28	1.41	-1.70	2.41
APOL2	1.13	1.32	-1.56	2.06
APOL6	2.16	2.19	1.15	1.90
APPBP2	-1.36	-1.29	-2.21	1.71
APPL2	-2.18	-2.76	-2.13	-1.30
AQR	-2.23	-2.74	-2.00	-1.37
ARAP2	3.70	3.06	1.94	1.58
ARAP3	-1.85	-2.18	-2.28	1.04
ARFIP1	-1.29	1.12	-3.33	3.74
ARG1	1.98	-1.40	3.54	-4.96
ARHGAP15	1.04	-1.12	-2.09	1.86
ARHGAP19	-2.72	-4.12	-2.16	-1.91
ARHGAP24	2.04	1.27	1.44	-1.13
ARHGAP25	-2.51	-1.83	-3.00	1.64
ARID3B	-1.63	-2.15	-1.48	-1.45
ARID4A	1.00	-1.40	-2.11	1.50
ARID4B	1.81	1.61	-1.68	2.71
ARID5A	2.47	2.02	1.92	1.05
ARL2BP	-2.45	-2.59	-2.07	-1.25
ARL5B	3.23	3.89	1.46	2.66
ARL6IP1	-2.59	-2.64	-1.99	-1.33

ARNT	-1.49	-2.10	-1.40	-1.50
ARNTL	-1.40	-1.80	-2.26	1.25
ARPP19	-2.27	-2.81	-2.65	-1.06
ARRB1	-2.76	-1.24	-6.33	5.12
ARRDC3	-1.56	-1.42	-2.85	2.01
ARSB	1.90	2.95	1.15	2.57
ARSG	-1.62	-1.71	-2.14	1.26
ASF1B	-3.75	-2.71	-5.31	1.96
ASH2L	-1.76	-2.05	-1.67	-1.23
ASPH	-1.62	-2.01	-2.03	1.01
ATAD2B	2.09	-1.06	1.01	-1.08
ATE1	-1.73	-2.25	-1.63	-1.38
ATF7IP	-2.26	-2.40	-2.18	-1.10
ATG2B	-1.73	-2.16	-2.07	-1.04
ATG7	2.69	3.73	1.06	3.52
ATHL1	-1.92	-1.28	-2.83	2.21
ATL2	-1.65	-1.86	-2.07	1.11
ATMIN	-3.14	-2.89	-2.23	-1.30
ATP11B	2.08	1.02	1.53	-1.50
ATP13A3	1.87	2.35	1.00	2.34
ATP1A1	-2.10	1.04	-3.13	3.24
ATP2B1	-4.87	-1.65	-11.02	6.68
ATP2B4	-1.25	-2.06	-1.48	-1.39
ATP2C2	2.12	1.42	2.95	-2.08
ATP5B	-1.88	-2.03	-1.45	-1.40
ATP5L	-1.66	-2.01	-2.03	1.01
ATP6V0E1	1.27	-1.38	-2.22	1.61
ATP6V1A	-2.79	-2.15	-4.53	2.11
ATP6V1C1	2.30	1.71	-1.03	1.76
ATP6V1D	-1.48	-1.41	-2.02	1.43
ATP6V1G1	-1.44	-1.06	-2.11	1.99
ATP7A	-2.92	-3.12	-3.21	1.03
AVIL	-1.45	-1.31	-2.40	1.83
AZI2	2.14	1.50	1.43	1.05
AZIN1	2.93	3.32	1.59	2.09
B3GNT5	4.04	3.69	1.97	1.87
B4GALT5	2.12	2.03	1.79	1.13
BACH1	-1.15	1.15	-1.95	2.24
BAG5	-2.05	-1.76	-1.82	1.03
BAT1	-1.45	-1.97	-2.72	1.38
BAT2L	-1.75	-2.29	-1.73	-1.32
BATF	2.99	3.11	2.16	1.44
BATF3	1.83	2.01	1.41	1.42
BAZ2B	-1.36	-1.93	-2.65	1.37
BBX	-1.23	-1.77	-2.04	1.15
BCKDK	-1.92	-1.45	-2.13	1.47
BCL2L11	1.77	2.01	-1.57	3.15
BCLAF1	1.04	-1.18	-2.33	1.98
BET1L	-2.04	-2.10	-2.06	-1.02
BHLHE40	-2.20	1.01	-3.39	3.42

BICD2	-2.65	-2.98	-2.13	-1.40
BIRC3	4.15	4.16	1.75	2.38
BMP2K	-1.03	-1.60	-2.18	1.37
BMX	1.30	-1.65	2.02	-3.33
BOD1L	-1.40	-1.57	-2.47	1.57
BPI	2.49	1.50	5.30	-3.53
BRCC3	-3.02	-3.60	-2.38	-1.51
BRI3BP	-2.42	-2.15	-2.13	-1.01
BRP44	-2.21	-2.31	-2.70	1.17
BRWD1	-2.02	-2.10	-2.59	1.23
BTBD1	-1.69	-2.03	-1.67	-1.22
BTF3L4	-3.50	-3.29	-3.31	1.01
BTK	-1.48	-2.60	-1.36	-1.91
BTN2A1	-1.30	-1.12	-2.28	2.04
BTN2A2	1.04	1.56	-1.60	2.50
BTN3A1	-2.15	-1.70	-3.60	2.13
BTN3A2	-2.46	-1.71	-4.64	2.71
BTN3A3	-2.12	-1.56	-3.15	2.02
BUB3	-2.00	-2.10	-1.90	-1.11
BXDC1	-1.14	2.56	-1.22	3.11
C10orf11	-2.31	-2.94	-2.94	-1.00
C10orf119	-1.65	-1.68	-2.08	1.23
C10orf137	-1.89	-2.03	-2.10	1.04
C10orf55	1.38	2.60	1.26	2.06
C10orf57	1.20	-1.29	2.02	-2.61
C11orf51	1.14	-1.41	1.52	-2.14
C11orf54	-1.83	-2.27	-2.30	1.01
C11orf75	2.38	3.86	-1.01	3.90
C11orf82	1.05	-1.27	1.75	-2.22
C12orf35	-1.25	-1.26	-2.62	2.07
C12orf4	2.99	2.32	1.82	1.27
C12orf5	-2.92	-2.91	-2.43	-1.20
C13orf23	5.19	1.71	5.14	-3.01
C13orf31	2.77	2.10	1.51	1.39
C14orf101	2.91	1.74	2.29	-1.32
C14orf106	-2.51	-2.86	-3.13	1.10
C14orf129	-1.93	-2.93	-1.78	-1.65
C14orf135	-2.20	-2.40	-2.24	-1.07
C14orf156	-2.71	-2.84	-2.27	-1.25
C14orf159	-2.00	-1.71	-3.30	1.92
C14orf2	-1.35	-2.12	-1.46	-1.45
C15orf24	-2.23	-2.00	-2.38	1.19
C15orf48	1.37	4.29	1.11	3.86
C16orf54	-2.18	-2.73	-1.80	-1.52
C16orf61	-1.85	-1.63	-2.01	1.23
C16orf63	-2.13	-2.06	-2.37	1.15
C16orf70	-2.20	-2.59	-1.79	-1.45
C16orf80	-1.93	-1.89	-2.03	1.08
C17orf63	2.57	2.42	1.73	1.39
C17orf65	-1.97	-2.49	-1.78	-1.40

C18orf25	-1.30	-1.59	-2.09	1.31
C18orf8	-2.16	-2.18	-2.05	-1.07
C19orf42	-2.18	-2.41	-2.08	-1.16
C19orf59	2.65	1.64	2.43	-1.48
C19orf61	2.72	2.92	1.41	2.07
C1orf103	-1.05	1.20	-2.58	3.09
C1orf128	-2.06	-2.35	-2.25	-1.04
C1orf162	1.16	-1.68	2.21	-3.73
C1orf183	-4.00	-3.73	-4.07	1.09
C1orf27	1.11	1.13	-1.93	2.17
C1orf43	-1.68	-1.17	-2.82	2.41
C1orf55	-2.31	-1.49	-3.00	2.02
C20orf11	-1.98	-2.00	-2.12	1.06
C20orf111	-1.18	-1.19	-2.35	1.98
C20orf112	-1.48	-1.45	-2.00	1.38
C20orf177	-1.14	-2.09	1.09	-2.27
C20orf19	-2.27	-2.81	-2.65	-1.06
C20orf20	-2.14	-1.90	-2.07	1.09
C20orf72	-2.56	-2.18	-2.18	-1.00
C20orf74	2.13	1.56	1.71	-1.09
C21orf91	3.21	3.49	1.44	2.41
C22orf28	1.26	-1.62	1.54	-2.49
C22orf30	-1.89	-2.04	-1.95	-1.05
C22orf9	-1.27	-1.84	1.09	-2.00
C2CD2L	-2.93	-2.47	-2.33	-1.06
C2orf24	-2.65	-3.40	-1.64	-2.07
C3	3.26	7.77	2.07	3.75
C3AR1	8.22	9.67	6.28	1.54
C3orf17	-1.36	-1.31	-2.09	1.60
C3orf34	-2.18	-1.80	-4.07	2.26
C3orf62	-2.14	-1.89	-3.02	1.60
C3orf63	-1.57	-2.50	-2.00	-1.25
C4BPA	1.52	1.25	-1.72	2.15
C4orf14	-2.15	-1.98	-2.09	1.05
C4orf16	2.39	1.86	1.48	1.26
C4orf29	-1.47	-1.59	-2.62	1.64
C4orf3	2.47	1.39	-1.82	2.52
C4orf33	-2.06	-1.73	-2.48	1.43
C4orf41	-1.46	-2.01	-1.78	-1.13
C5AR1	-2.28	-2.60	-1.73	-1.50
C5orf32	2.49	1.93	1.80	1.07
C5orf36	-1.51	-2.00	-2.69	1.34
C5orf41	1.10	1.17	-2.14	2.50
C6orf115	-2.08	-2.04	-2.59	1.27
C6orf150	5.59	4.39	2.21	1.99
C6orf204	-3.37	-4.05	-4.42	1.09
C6orf211	1.72	1.74	-1.24	2.15
C6orf62	-1.11	1.09	-2.22	2.42
C6orf72	-2.33	-2.36	-4.84	2.06
C7orf23	-3.32	-3.20	-3.59	1.12

C7orf25	-2.18	-2.91	-2.23	-1.31
C7orf41	-1.77	-2.05	-2.17	1.06
C7orf43	-2.73	-2.90	-2.39	-1.21
C7orf44	-1.58	-2.07	-2.36	1.14
C7orf60	1.06	1.21	-2.08	2.52
C7orf70	-2.04	-1.50	-1.99	1.33
C9orf72	1.15	1.14	-2.30	2.61
C9orf91	-1.46	1.71	-1.77	3.02
CABC1	-2.27	-2.08	-2.42	1.17
CACNB4	-2.34	-1.86	-2.42	1.30
CALM2	-2.06	-2.58	-4.62	1.79
CAMK1G	1.82	2.03	1.27	1.61
CAMK2G	-2.79	-3.76	-2.41	-1.56
CAMKK2	-2.48	-3.01	-2.20	-1.37
CAPG	1.76	1.16	2.37	-2.06
CAPN1	-1.00	-1.60	1.26	-2.02
CAPN7	-2.03	-2.55	-2.80	1.10
CAPNS1	-1.14	-1.78	1.24	-2.22
CAPZA2	-1.05	-1.01	-2.04	2.02
CARD8	-1.73	-1.77	-2.03	1.15
CARHSP1	-1.99	-2.72	-1.59	-1.71
CASP1	1.37	1.42	-1.74	2.46
CASP10	1.96	3.04	-1.12	3.39
CASP2	-1.71	-2.41	-1.49	-1.62
CASP5	2.05	3.47	-1.75	6.07
CASP8	-1.64	-1.60	-2.38	1.49
CASS4	-4.12	-1.82	-6.51	3.57
CBFB	-1.78	-2.11	-1.59	-1.32
CBWD3	-1.36	-1.86	-2.30	1.24
CBX3	1.13	-1.46	-2.07	1.42
CBX7	-2.31	-2.64	-2.75	1.04
CCBL2	1.33	1.09	-1.88	2.06
CCDC109A	-1.70	-2.37	-2.16	-1.10
CCDC125	-2.99	-3.52	-2.81	-1.25
CCDC144NL	-1.64	-2.24	-1.80	-1.24
CCDC146	-2.01	-2.02	-3.79	1.88
CCDC69	-1.71	-2.06	-1.98	-1.04
CCDC84	-2.07	-1.76	-1.97	1.12
CCL20	1.87	2.40	1.30	1.84
CCL23	1.16	2.73	1.10	2.49
CCL4	1.60	4.69	1.11	4.23
CCM2	-2.04	-2.44	-1.96	-1.25
CCNL1	1.99	2.16	1.03	2.10
CCNY	-1.92	-1.75	-2.41	1.38
CCPG1	-2.13	-2.83	-2.35	-1.21
CCR1	1.41	1.76	-1.73	3.04
CCR3	-4.81	-2.89	-5.42	1.87
CCRL2	2.65	7.63	1.39	5.48
CD14	2.05	1.90	1.61	1.18
CD177	7.48	3.66	16.15	-4.41

CD24	1.99	1.84	2.72	-1.48
CD27	-1.93	-2.46	-2.20	-1.12
CD274	5.28	9.03	-1.08	9.74
CD300A	-3.13	-1.98	-3.85	1.94
CD300LB	-3.94	-3.60	-3.96	1.10
CD300LF	-2.99	-3.04	-2.53	-1.20
CD33	-2.41	-2.36	-2.21	-1.07
CD3D	-2.26	-2.45	-2.58	1.05
CD3E	-2.46	-3.13	-3.20	1.02
CD3G	-4.13	-4.86	-4.69	-1.04
CD4	-2.39	-1.66	-2.34	1.41
CD40	1.72	2.09	1.20	1.74
CD44	3.39	3.25	2.80	1.16
CD46	-1.22	-1.38	-2.36	1.71
CD47	-1.77	-1.86	-2.62	1.41
CD48	2.98	3.22	2.17	1.48
CD52	-2.31	-2.65	-2.34	-1.13
CD59	2.24	1.82	1.68	1.08
CD68	-1.70	-1.40	-2.42	1.73
CD69	2.55	9.22	1.22	7.54
CD74	-1.55	-1.13	-2.26	1.99
CD83	1.24	2.54	1.00	2.53
CD84	-1.69	1.67	-1.63	2.71
CD93	-2.63	-2.43	-2.63	1.08
CD96	-1.95	-2.23	-2.25	1.01
CDADC1	4.25	1.90	3.54	-1.87
CDC2L5	-1.71	-2.06	-1.77	-1.16
CDC2L6	-1.63	-2.29	-2.18	-1.05
CDC40	-2.10	-2.49	-1.78	-1.40
CDKL5	-1.61	-2.39	-1.71	-1.40
CDKN1A	1.84	2.81	1.11	2.53
CDKN1B	-1.85	-1.87	-2.17	1.16
CDS2	-2.46	-2.50	-3.64	1.45
CDV3	-1.93	-2.41	-1.73	-1.39
CEACAM1	3.86	2.87	3.96	-1.38
CEP170	-4.73	-3.58	-4.82	1.35
CEP63	-6.28	-4.30	-10.36	2.41
CEP97	-3.11	-3.32	-2.32	-1.43
CEPT1	2.19	2.04	1.33	1.54
CFD	-1.99	-1.55	-2.51	1.62
CFL2	8.24	8.75	2.06	4.26
CHCHD7	2.25	1.59	1.66	-1.05
CHD1	2.16	1.82	-1.25	2.28
CHD2	1.57	1.59	-1.26	2.00
CHD9	-1.90	-2.04	-1.93	-1.05
CHI3L1	-1.30	-1.04	-3.35	3.21
CHI3L2	2.63	2.64	1.35	1.95
CHMP1B	-1.95	-1.92	-2.10	1.09
CHMP5	1.10	1.20	-1.76	2.11
CHORDC1	2.06	1.30	1.99	-1.53

CHPT1	-1.23	-2.02	-1.06	-1.90
CHURC1	-1.15	-1.19	-2.38	2.00
CIDEB	-3.12	-4.36	-3.15	-1.39
CIITA	1.19	2.30	-1.08	2.49
CIR1	-1.13	1.09	-3.03	3.30
CIRBP	-1.84	-1.79	-2.14	1.20
CISH	3.40	3.93	2.37	1.65
CITED2	-1.94	-1.65	-2.24	1.35
CLC	3.41	1.08	6.50	-6.01
CLCN4	-1.67	-1.21	-2.21	1.83
CLEC12A	-1.00	-1.63	1.33	-2.16
CLEC2B	-1.16	1.08	-3.08	3.33
CLEC4D	7.69	6.04	6.32	-1.05
CLEC5A	4.20	4.21	5.13	-1.22
CLEC7A	-2.60	-1.71	-6.98	4.09
CLEC9A	-3.09	-2.56	-3.12	1.22
CLIC4	21.26	23.42	11.02	2.13
CLK1	1.04	1.00	-2.31	2.32
CLN6	-3.19	-2.25	-3.14	1.40
CLPP	-2.41	-2.44	-2.37	-1.03
CMAH	2.41	1.83	1.60	1.15
CMTM7	-1.85	-2.21	-1.77	-1.25
CNIH4	3.05	1.89	2.06	-1.09
CNOT10	-2.20	-2.21	-2.51	1.13
CNOT6	-1.24	-2.01	-1.48	-1.36
CNOT7	-2.34	-2.72	-3.03	1.11
COG1	-2.17	-2.26	-2.10	-1.07
COMMD8	-2.29	-2.89	-2.95	1.02
COPS3	-4.72	-4.46	-4.44	-1.00
COPS6	-2.00	-2.30	-1.91	-1.20
COQ10B	1.08	1.12	-1.84	2.06
CORO1B	-2.31	-2.08	-2.42	1.17
CORO1C	-3.01	-3.63	-3.52	-1.03
CP110	-1.33	-2.37	-1.35	-1.76
CPEB2	-2.46	-2.50	-3.06	1.22
CPPED1	-2.27	-1.98	-2.79	1.41
CPT2	-1.98	-2.24	-1.87	-1.19
CREB1	-1.13	-1.04	-2.17	2.08
CREBL2	1.27	2.60	1.05	2.48
CREG1	1.11	1.47	-1.74	2.56
CRIPT	-1.59	-1.75	-2.18	1.25
CRISP3	6.98	2.80	16.92	-6.04
CRLF3	-2.31	-3.12	-2.41	-1.29
CRTC3	2.43	1.45	2.76	-1.90
CSDA	-2.05	-1.36	-3.18	2.34
CSE1L	-2.38	-2.61	-2.90	1.11
CSF1R	-1.91	-1.68	-2.91	1.73
CSNK1G3	-1.28	-1.39	-2.06	1.48
CSNK2A1	-1.77	-2.20	-1.29	-1.71
CSRNP1	2.54	2.96	1.43	2.07

CSTA	1.50	1.35	-1.78	2.40
CSTF1	-1.67	-1.44	-2.07	1.43
CTBS	-1.24	-1.31	-2.24	1.71
CTCF	-3.06	-2.91	-2.54	-1.14
CTD-2514C3.1	1.73	1.69	2.05	-1.21
CTDSPL2	-2.16	-2.22	-3.40	1.53
CTSC	-3.03	-1.86	-4.28	2.30
CTSH	10.42	6.13	11.07	-1.80
CUGBP2	-1.65	-2.11	-1.73	-1.22
CUL4B	-1.23	-1.68	-2.04	1.21
CX3CR1	-1.18	-1.25	-2.27	1.82
CXCL10	6.96	33.78	2.29	14.74
CXCL11	1.28	2.67	1.26	2.11
CXCL16	1.24	1.57	-1.36	2.13
CXCR4	-4.14	-1.68	-2.59	1.54
CXorf21	1.44	1.68	-1.98	3.33
CXorf38	-1.68	-1.40	-2.64	1.89
CYB5B	-1.76	-2.50	-1.37	-1.83
CYB5D1	2.23	1.70	1.64	1.04
CYB5R4	-1.01	-1.05	-2.13	2.03
CYBB	2.96	3.10	3.32	-1.07
CYBRD1	-2.53	-3.04	-4.20	1.38
CYP27A1	-1.37	1.15	-3.34	3.83
CYSLTR1	-2.75	-2.40	-3.98	1.66
CYTSA	3.69	1.80	3.51	-1.95
DAD1	-2.15	-2.38	-1.88	-1.27
DAP	-1.63	-1.32	-2.41	1.82
DAPK1	-1.79	-1.49	-2.44	1.64
DAPP1	2.13	2.21	-1.38	3.05
DCAF16	-1.70	-1.81	-2.09	1.16
DCAF5	-1.55	-2.13	-1.37	-1.56
DCAF7	-1.96	-2.05	-1.50	-1.36
DCUN1D3	3.75	4.30	2.18	1.97
DDIT3	-1.72	-1.59	-2.35	1.47
DDX20	-2.45	-2.38	-2.27	-1.05
DDX21	1.85	2.54	1.07	2.36
DDX46	-1.70	-2.23	-2.01	-1.11
DDX58	2.11	2.13	-1.22	2.59
DDX60	2.05	2.55	-1.44	3.66
DECR1	-1.52	-1.29	-2.81	2.18
DEF6	-1.85	-2.20	-1.77	-1.24
DEK	-1.07	-1.21	-2.26	1.87
DENND2D	2.70	2.35	2.00	1.18
DENND4A	1.67	3.28	-1.47	4.82
DGCR2	-1.73	-2.24	-1.59	-1.41
DGKA	-1.64	-2.37	-1.26	-1.89
DGKD	-1.61	-2.35	-1.27	-1.85
DHCR7	1.46	1.01	3.14	-3.12
DHDDS	-2.31	-2.02	-2.00	-1.01
DHRS7	-1.79	-2.05	-2.22	1.09

DHTKD1	-1.77	-2.07	-1.83	-1.13
DHX29	-1.76	-2.02	-1.84	-1.10
DHX34	-1.85	-2.11	-1.75	-1.21
DHX9	-1.32	-1.69	-2.02	1.19
DIAPH2	2.11	1.46	1.46	-1.00
DICER1	-2.29	-2.19	-3.26	1.49
DLEU2	-1.07	-1.28	-2.12	1.65
DMXL2	2.77	3.24	1.45	2.23
DNAJA1	2.41	2.49	1.42	1.75
DNAJB6	-1.34	-1.01	-2.26	2.23
DNAJB9	1.32	2.19	-1.10	2.40
DNAJC13	-1.51	-2.17	-1.75	-1.24
DNAJC3	-1.95	-1.42	-3.57	2.51
DNAJC9	-1.65	-2.05	-1.50	-1.36
DOCK11	-1.27	-2.14	-1.94	-1.10
DOCK4	1.40	1.93	-1.88	3.63
DOCK5	-1.76	-1.74	-3.09	1.78
DOCK8	-1.69	-2.01	-2.06	1.03
DOT1L	1.77	2.11	1.47	1.44
DPEP2	-3.39	-3.33	-4.08	1.23
DPEP3	-3.62	-3.51	-3.89	1.11
DPH2	-1.82	-1.68	-2.10	1.25
DPY19L3	-1.25	-2.21	-1.02	-2.16
DPY30	-3.61	-4.67	-3.60	-1.30
DSC2	-1.68	-1.26	-4.75	3.77
DSCR3	-2.59	-3.18	-2.07	-1.54
DSN1	-2.91	-2.83	-2.69	-1.05
DTX3L	1.62	1.73	-1.26	2.18
DULLARD	-2.10	-1.77	-1.58	-1.12
DUS2L	-3.11	-3.69	-2.32	-1.59
DUSP18	-1.92	-2.32	-1.75	-1.33
DUSP3	2.39	2.44	1.41	1.73
DUSP5	1.92	3.81	1.22	3.13
DUSP6	-3.14	-1.43	-3.65	2.55
DUSP7	-3.15	-2.88	-3.36	1.17
DYNC1I2	1.50	1.09	2.48	-2.27
DYNC1LI1	-3.05	-2.59	-4.11	1.58
DYNLL1	-2.21	-2.44	-2.20	-1.11
DYNLT3	3.66	4.33	2.40	1.81
EBI3	1.32	2.08	1.18	1.77
ECT2	2.54	1.52	1.21	1.26
EDEM1	-1.93	-1.65	-2.53	1.54
EEPD1	-1.70	-1.90	-2.61	1.38
EFCAB2	-1.62	-1.87	-2.35	1.25
EFTUD2	-2.70	-2.63	-2.16	-1.22
EGR1	1.80	1.73	2.04	-1.18
EGR3	1.51	2.82	1.23	2.30
EHD1	2.69	2.71	2.11	1.28
EIF1AY	-3.44	-3.04	-4.98	1.64
EIF1B	1.69	2.11	1.03	2.05

EIF2AK1	-1.59	-2.06	-1.53	-1.35
EIF2AK2	2.77	2.19	1.23	1.78
EIF2C1	-1.93	-2.06	-2.32	1.13
EIF3D	-1.79	-1.33	-2.01	1.51
EIF3J	-1.65	-1.68	-2.02	1.20
EIF3L	-2.54	-2.93	-2.02	-1.45
EIF3M	-3.83	-4.84	-4.02	-1.20
EIF4A1	-1.94	-2.09	-2.13	1.02
EIF4ENIF1	-2.03	-2.10	-1.85	-1.13
EIF4H	-1.23	-1.29	-2.17	1.69
EIF5	-1.47	-1.38	-2.07	1.50
ELF2	1.34	1.23	-1.67	2.06
ELL2	1.24	1.50	-1.39	2.09
ELMO2	2.36	1.38	1.68	-1.22
ELMOD2	-1.71	-2.10	-1.96	-1.07
ELOF1	-2.07	-2.26	-1.83	-1.23
EMR1	11.00	9.41	10.19	-1.08
EMR2	-1.93	-1.28	-3.16	2.48
ENPP2	2.36	1.40	1.53	-1.09
ENTPD7	2.86	1.59	5.07	-3.18
EPAS1	2.09	1.52	1.79	-1.18
EPB41	-1.55	-1.82	-2.63	1.45
EPHB1	-2.60	-1.87	-3.35	1.80
EPM2AIP1	1.99	2.23	1.02	2.19
EPS15L1	-3.07	-3.78	-3.70	-1.02
EPSTI1	1.90	2.13	-1.41	3.01
ERAL1	2.03	2.24	1.73	1.29
ERAP1	-1.80	-1.43	-2.34	1.63
ERBB2IP	-1.08	1.12	-2.51	2.80
ERCC5	-1.67	-2.02	-1.56	-1.29
ERCC6	-2.11	-2.19	-2.13	-1.03
ERGIC1	-1.32	-1.27	-2.26	1.77
ERH	-2.16	-2.21	-2.27	1.03
ERI1	3.46	2.15	1.49	1.44
ERLIN1	2.29	1.59	1.96	-1.23
ERN1	-1.87	-1.84	-2.48	1.35
ERO1L	-1.87	-2.82	-1.53	-1.84
ERP27	-3.41	-4.01	-3.05	-1.31
ERV3	-1.48	-1.55	-9.26	5.97
ESCO1	-1.85	-1.67	-2.91	1.74
ESYT2	-1.85	-1.72	-2.22	1.29
ETS2	2.74	2.69	2.04	1.32
ETV7	1.39	2.19	-1.28	2.81
EVI2A	-2.91	-2.38	-5.50	2.31
EVI5	-1.35	-2.25	-2.03	-1.11
EXOSC1	-2.14	-2.35	-1.92	-1.23
EXOSC4	1.95	1.74	2.42	-1.39
EXOSC9	-1.15	1.26	-1.59	2.01
EXTL3	-1.49	-1.67	-2.13	1.27
EYA3	1.54	2.17	-1.27	2.76

EZH2	2.03	2.43	1.17	2.07
F11R	-1.91	-1.21	-2.97	2.45
F2RL1	-2.38	-2.17	-3.42	1.58
F5	2.44	1.33	1.77	-1.33
F8A1	-1.83	-2.06	-1.41	-1.46
FADD	-1.81	-2.14	-1.40	-1.53
FAIM3	-2.55	-2.40	-2.88	1.20
FAM111A	-1.59	-1.60	-2.68	1.68
FAM116A	-1.99	-2.29	-2.69	1.17
FAM117B	-3.37	-3.70	-3.97	1.07
FAM127A	1.32	-1.88	1.09	-2.05
FAM134A	-2.16	-2.24	-1.89	-1.19
FAM174A	-3.05	-2.62	-6.18	2.36
FAM177A1	3.09	1.80	1.42	1.27
FAM188A	2.15	1.65	1.02	1.61
FAM190B	-2.07	-2.09	-1.88	-1.11
FAM36A	-3.37	-3.89	-4.60	1.18
FAM49A	-1.54	-1.17	-3.37	2.88
FAM53B	-2.05	-1.70	-2.36	1.39
FAM54B	-1.98	-2.13	-1.89	-1.13
FAM63A	-2.12	-2.83	-1.87	-1.52
FAM63B	-2.06	-2.23	-1.63	-1.37
FAM78A	-2.07	-1.90	-1.98	1.04
FAM83E	-2.76	-3.09	-2.00	-1.54
FAM91A1	-1.57	-1.50	-3.04	2.03
FAM96A	-1.97	-2.12	-2.20	1.04
FANCE	-2.76	-3.46	-3.11	-1.11
FAR1	-1.18	-2.75	-1.27	-2.18
FAR2	-2.77	-3.16	-1.88	-1.68
FAS	1.78	1.74	-1.47	2.55
FBN2	1.16	1.44	-1.71	2.46
FBXL13	-1.08	-1.13	-3.20	2.83
FBXL20	-2.64	-3.81	-2.99	-1.27
FBXL4	-1.93	-2.23	-1.91	-1.17
FBXO9	2.10	1.06	1.69	-1.59
FCAR	2.32	1.65	2.24	-1.36
FCGR1A	1.57	1.72	-1.38	2.37
FCGR2B	-2.69	-1.76	-4.18	2.38
FCHO1	-2.26	-2.52	-2.33	-1.08
FCHO2	-1.05	-1.89	-3.54	1.88
FCRL1	2.53	1.29	3.75	-2.91
FDFT1	-1.48	-2.13	-1.44	-1.48
FFAR2	1.97	2.04	1.47	1.39
FFAR3	2.30	2.31	1.24	1.86
FGD4	2.41	1.88	1.61	1.16
FGFR1OP	-2.13	-1.96	-2.37	1.21
FGL2	-2.83	-2.44	-4.72	1.94
FHL3	-3.15	-2.64	-3.20	1.22
FIG4	-1.82	-2.17	-1.95	-1.11
FKBP11	2.23	1.92	1.59	1.21

FKBP5	2.45	1.89	1.66	1.14
FLII	-1.54	-2.19	-1.47	-1.50
FLJ10213	2.32	1.58	1.26	1.26
FLJ36031	1.76	2.06	1.13	1.83
FLJ43826	-1.94	-2.02	-2.12	1.05
FLOT1	2.16	2.27	1.45	1.56
FLT3LG	-1.58	-1.54	-2.08	1.35
FLVCR1	1.01	1.10	-2.06	2.26
FMNL3	2.58	6.53	1.44	4.55
FNBP1	-1.23	1.57	-1.50	2.36
FNBP4	-2.78	-2.44	-2.70	1.11
FNDC3A	3.57	2.69	1.80	1.49
FNDC3B	2.00	2.25	1.09	2.08
FNTA	-1.08	-1.68	-2.14	1.28
FNTB	-2.13	-1.93	-1.90	-1.01
FOXN2	-1.91	-3.10	-1.59	-1.95
FOXN3	-1.99	-2.38	-2.25	-1.06
FRAT1	-2.32	-2.57	-2.60	1.01
FRAT2	-2.07	-2.29	-2.10	-1.09
FRG1	-2.01	-1.89	-2.18	1.16
FRY	-2.18	-3.17	-2.88	-1.10
FRYL	-1.45	-2.17	-2.22	1.02
FSCN1	1.15	3.33	-1.07	3.57
FTSJD2	1.34	1.68	-1.36	2.29
FYB	-1.31	-1.30	-2.26	1.74
FYN	-1.99	-2.24	-2.33	1.04
G0S2	2.62	2.64	1.76	1.50
G3BP1	-2.02	-1.83	-1.98	1.08
GAB1	-2.25	-2.92	-3.85	1.32
GABBR1	2.43	1.38	3.13	-2.27
GABPB1	1.68	2.06	-1.16	2.39
GADD45B	1.89	2.21	1.33	1.66
GALC	2.31	2.77	1.36	2.04
GALM	-2.38	-2.63	-2.59	-1.02
GALNT3	-2.79	-3.19	-2.24	-1.43
GARNL1	3.52	3.11	1.31	2.38
GARNL4	-1.87	-2.30	-1.92	-1.20
GAS7	2.52	2.00	2.48	-1.24
GATC	-3.08	-3.33	-1.91	-1.75
GBP1	2.84	3.57	-1.18	4.21
GBP3	3.10	4.34	1.09	3.97
GBP4	1.71	3.84	-3.10	11.93
GBP5	1.28	1.88	-3.37	6.33
GBP7	1.20	1.49	-2.75	4.09
GCC2	1.00	-1.22	-2.03	1.67
GCH1	18.80	17.51	9.96	1.76
GCM1	-3.12	-2.52	-3.08	1.22
GDE1	-2.25	-3.14	-2.59	-1.21
GDI2	-1.21	-1.77	-2.08	1.18
GIMAP2	-1.72	1.05	-3.94	4.14

GIMAP4	-1.57	-1.06	-3.98	3.77
GIMAP7	-1.92	-2.10	-2.13	1.02
GJB6	1.69	1.24	2.28	-1.84
GK	2.31	2.34	1.27	1.85
GK3P	2.60	1.73	1.72	1.01
GK5	2.01	1.14	1.92	-1.69
GLA	-1.15	1.32	-1.82	2.40
GLB1	-2.24	-2.80	-2.81	1.00
GLRX	1.62	2.11	-1.33	2.80
GLT8D1	-3.74	-3.55	-3.57	1.01
GLUD1	-1.63	-2.61	-1.98	-1.32
GLUL	-1.38	-1.23	-2.21	1.80
GMCL1	-1.85	-2.17	-1.84	-1.18
GMFB	-2.23	-2.32	-1.79	-1.30
GNA15	2.99	2.71	1.93	1.40
GNB2L1	-1.51	-2.36	-1.23	-1.91
GNLY	-1.97	-2.11	-2.18	1.03
GOLPH3L	-1.84	-1.48	-2.37	1.61
GORASP2	-1.18	1.35	-1.94	2.62
GPBP1	1.27	1.41	-1.65	2.32
GPI	1.00	-1.21	2.06	-2.49
GPR107	-1.03	1.38	-1.61	2.22
GPR132	2.55	2.08	2.39	-1.15
GPR141	6.98	4.81	4.35	1.11
GPR155	1.09	-1.44	-2.01	1.39
GPR162	-2.69	-2.02	-2.60	1.29
GPR174	-2.19	-2.99	-2.78	-1.07
GPR177	-2.87	-2.49	-4.64	1.87
GPR19	-2.15	-2.12	-2.02	-1.05
GPR65	2.41	2.34	1.74	1.35
GPR77	-3.34	-4.17	-2.95	-1.41
GPR84	23.99	23.47	21.64	1.08
GRAMD1A	2.71	2.80	2.18	1.28
GRAMD1C	-7.31	-5.44	-7.60	1.40
GRINA	3.06	3.13	2.61	1.20
GSK3B	-1.49	-1.93	-2.24	1.16
GSR	-2.84	-3.04	-2.68	-1.13
GTF2A1	-1.83	-1.66	-2.33	1.40
GTF2B	1.13	1.39	-1.46	2.04
GTPBP1	2.27	1.92	2.06	-1.07
GTPBP10	-2.26	-2.28	-2.10	-1.08
GYG1	6.70	3.50	5.72	-1.64
GYS1	1.30	-1.04	2.03	-2.11
GZMK	-2.43	-2.81	-2.88	1.02
H2AFZ	-1.76	-2.37	-2.06	-1.15
HADH	-1.83	-1.90	-2.16	1.14
HAGH	-1.98	-2.01	-1.60	-1.25
HAL	-1.65	-2.09	-2.87	1.37
HBP1	1.04	1.09	-1.99	2.17
HCG27	-1.75	-1.62	-2.31	1.43

HCP5	1.06	1.14	-1.80	2.05
HDAC1	-2.48	-3.24	-2.31	-1.40
HDAC2	1.59	1.96	-1.18	2.31
HDAC3	-2.50	-2.86	-2.23	-1.29
HDAC5	-1.78	-1.69	-2.10	1.24
HDGF	1.98	3.30	-1.07	3.53
HEATR5A	-1.69	-3.65	-3.60	-1.02
HEBP1	-2.78	-2.74	-3.39	1.24
HELB	9.85	7.64	4.35	1.76
HERC1	-1.81	-2.70	-1.87	-1.45
HERC3	-1.38	-1.71	-2.12	1.24
HERC5	1.83	2.53	-2.12	5.36
HERC6	1.59	1.96	-1.44	2.81
HERPUD1	-2.42	-2.15	-2.41	1.12
HEXB	-2.01	-1.77	-3.92	2.21
HEXIM1	-2.53	-2.06	-2.74	1.33
HGF	4.87	1.69	14.00	-8.26
HGSNAT	-1.22	-2.02	-1.11	-1.81
HIATL1	-1.27	-2.02	1.01	-2.04
HIGD2A	-1.35	-1.86	-2.23	1.20
HINT3	-1.16	-1.15	-2.33	2.03
HIRA	-2.19	-2.51	-1.90	-1.32
HISPPD1	-1.43	-2.22	-1.67	-1.32
HIST1H1C	-2.07	-2.49	-2.77	1.11
HIST1H1E	-1.89	-2.47	-1.71	-1.44
HIST1H2BC	-1.79	-2.23	-3.71	1.67
HIST1H2BD	-1.40	-2.19	-2.14	-1.02
HIST1H2BF	-1.89	-2.06	-2.24	1.09
HIST1H2BG	-1.97	-1.83	-2.17	1.19
HIST1H3A	-1.55	-1.73	-3.10	1.79
HIST1H3C	-1.53	-1.68	-2.20	1.31
HIST1H4C	-1.75	-2.37	-2.36	-1.00
HIST1H4E	-1.51	-1.74	-2.01	1.15
HIST1H4L	-1.69	-1.77	-2.04	1.15
HIST2H2BF	-1.93	-2.35	-5.16	2.20
HIST4H4	-2.03	-2.61	-2.13	-1.23
HIVEP1	3.35	3.38	1.62	2.09
HIVEP2	4.59	4.96	2.01	2.47
HK3	2.26	1.28	3.13	-2.45
HLA-DMA	-1.81	-1.41	-2.04	1.45
HLA-DPA1	-4.48	-5.36	-5.57	1.04
HLA-DPB1	-3.57	-4.11	-4.25	1.03
HLA-DRA	-2.39	1.33	-3.77	5.03
HLA-F	-1.01	1.16	-2.02	2.34
HLX	2.04	2.16	1.57	1.38
HMGB2	1.31	-1.60	1.48	-2.36
HMGCR	-1.63	-1.64	-2.20	1.34
HMGN4	-2.45	-2.07	-2.64	1.27
HMOX2	-3.11	-3.14	-2.18	-1.44
HNRNPA1	-1.21	-1.80	-2.10	1.16

HNRNPK	-1.24	-1.39	-2.14	1.54
HNRNPM	1.11	1.11	-1.87	2.07
HNRNPR	-1.76	-1.53	-2.63	1.72
HORMAD1	-1.20	-1.19	-2.39	2.00
HP	4.83	2.54	8.66	-3.40
HPGD	2.13	1.30	2.17	-1.67
HPR	1.87	1.40	3.17	-2.27
HPS3	-2.20	-2.82	-2.32	-1.21
HPSE	-3.47	-3.31	-4.20	1.27
HRH4	3.49	3.11	3.67	-1.18
HS3ST3B1	3.41	2.19	2.64	-1.20
HSD17B11	-1.36	-1.44	-2.17	1.50
HSD17B7P2	-2.12	-1.90	-1.72	-1.10
HSDL2	-1.33	-2.11	-1.79	-1.18
HSH2D	-1.71	-1.35	-3.00	2.23
HSP90AA1	2.37	2.25	1.27	1.76
HSP90B1	-2.09	1.16	-3.45	4.02
HSPA13	-1.00	2.22	-1.45	3.21
HSPA1B	2.12	2.11	1.29	1.64
HSPA4	-1.63	-1.32	-2.19	1.65
HSPBAP1	-2.20	-2.00	-2.68	1.34
HSPC159	-2.14	1.07	-4.47	4.80
HVCN1	-2.40	-2.62	-2.42	-1.08
ICAM1	2.47	2.79	1.25	2.23
ID2	-1.33	-1.72	-2.05	1.20
IDH1	-2.47	-2.44	-2.36	-1.04
IDI1	2.73	1.86	2.04	-1.09
IDO1	1.42	6.61	1.00	6.60
IDS	-1.80	-1.95	-2.06	1.05
IER3	2.14	2.49	1.74	1.43
IFI44	3.05	2.26	-1.11	2.51
IFI44L	2.57	2.04	-1.03	2.10
IFI6	1.07	1.36	-1.90	2.59
IFIH1	3.83	4.91	1.01	4.86
IFIT1	1.03	1.62	-3.11	5.05
IFIT2	1.05	1.50	-3.08	4.62
IFIT3	1.26	1.53	-1.81	2.77
IFIT5	1.59	1.85	-1.84	3.39
IFNAR2	-1.32	-1.59	-2.15	1.35
IFRD1	-1.59	-1.82	-2.42	1.33
IGJ	-2.14	-2.91	-3.13	1.07
IGK@	-1.58	-2.47	-2.84	1.15
IK	-1.41	-1.45	-2.03	1.40
IKBKB	1.31	1.55	-1.29	2.01
IKBKE	1.99	3.24	1.31	2.47
IKIP	-8.45	-8.37	-11.56	1.38
IL10RA	1.96	2.11	1.65	1.28
IL13RA1	1.16	1.14	-1.90	2.16
IL18R1	5.79	2.75	2.90	-1.05
IL1A	2.10	9.03	1.53	5.91

IL1B	2.48	3.44	1.09	3.16
IL1RN	2.84	3.11	1.73	1.80
IL4R	2.19	1.67	2.13	-1.28
IL6R	-2.05	-2.17	-2.16	-1.01
IL6ST	7.49	6.18	3.34	1.85
IL7R	-4.11	-6.28	-6.67	1.06
IL8	1.64	1.81	-1.32	2.39
ILF2	2.71	1.52	1.67	-1.09
IMPA2	-2.17	-3.08	-2.16	-1.42
INPP5B	-2.07	-1.93	-2.55	1.32
INPP5D	-1.74	-2.32	-2.14	-1.09
INSIG1	-2.38	-1.69	-3.14	1.86
INTS8	-1.71	-1.71	-2.53	1.48
IP6K1	-1.26	-1.24	-2.01	1.62
IPCEF1	-2.42	-2.62	-1.89	-1.39
IQGAP2	-1.73	-2.17	-2.53	1.17
IRAK2	3.93	6.14	2.19	2.81
IRAK3	4.77	4.18	3.43	1.22
ISCA1	-1.54	-2.08	-1.29	-1.61
ISCA2	-1.66	-2.21	-1.85	-1.19
ISG20	-1.18	1.38	-2.03	2.82
ISG20L2	-1.56	-1.42	-2.24	1.57
ITGA4	-3.20	-2.95	-3.25	1.10
ITGA5	-3.19	-2.70	-4.43	1.64
ITGA6	1.94	1.01	2.58	-2.56
ITGAL	-1.96	-2.42	-2.01	-1.21
ITPK1	-2.90	-2.78	-3.09	1.11
ITPKB	-1.97	-1.85	-2.06	1.11
IVNS1ABP	2.05	2.33	1.13	2.06
JAK2	2.42	1.38	1.61	-1.16
JARID2	-3.65	-3.08	-5.96	1.93
JDP2	-2.78	-2.75	-2.50	-1.10
JKAMP	-2.69	-3.48	-2.32	-1.50
JMJD1C	-1.72	-1.87	-3.20	1.71
JOSD1	2.90	1.92	2.50	-1.30
JTB	-1.83	-1.74	-2.17	1.25
KBTBD6	1.06	-1.02	2.03	-2.07
KBTBD7	-1.68	-2.50	1.11	-2.77
KCNAB2	-1.34	-2.15	-1.40	-1.54
KCNE3	-1.89	-2.18	-2.28	1.05
KCNJ2	1.39	2.01	-1.33	2.68
KCTD12	-2.01	-1.89	-1.96	1.04
KCTD20	-1.76	-1.09	-3.32	3.05
KDELR2	-2.24	-1.41	-2.19	1.55
KDM3A	-2.79	-2.53	-4.69	1.85
KHDRBS1	-1.03	-1.05	-2.02	1.91
KIAA0232	-2.28	-2.33	-2.66	1.14
KIAA0319L	-2.24	-2.49	-2.74	1.10
KIAA0391	-1.85	-2.31	-1.90	-1.22
KIAA0753	-2.83	-2.89	-2.63	-1.10

KIAA1191	-2.52	-2.84	-2.44	-1.16
KIAA1257	-2.09	-2.06	-3.15	1.53
KIAA1324	-1.83	-1.63	-4.69	2.88
KIAA1632	3.28	3.41	1.97	1.73
KIAA1737	-1.78	-1.64	-2.11	1.29
KIF13A	-1.77	-1.72	-3.09	1.79
KIF2A	-2.19	-2.82	-2.63	-1.07
KL	2.91	1.74	3.05	-1.75
KLF10	-1.29	1.63	-1.37	2.23
KLF13	-1.95	-2.35	-1.38	-1.70
KLF3	-4.00	-4.58	-4.42	-1.04
KLHDC2	-3.37	-3.62	-3.96	1.09
KLHDC3	-1.75	-2.11	-1.66	-1.27
KLHDC8B	-3.53	-2.13	-2.80	1.32
KLHL21	-2.14	-2.07	-2.30	1.11
KLHL24	-1.73	-1.69	-3.17	1.87
KLHL5	2.11	2.09	1.05	1.99
KLRB1	-1.78	-1.90	-2.10	1.11
KLRD1	-1.90	-1.91	-2.13	1.12
KLRK1	-2.06	-2.49	-2.57	1.03
KPNA3	-2.32	-2.68	-2.22	-1.20
LACTB	3.36	3.06	1.88	1.63
LAIR1	4.65	3.39	3.80	-1.12
LAMP3	2.35	3.94	1.09	3.63
LASS2	-3.26	-3.66	-2.98	-1.23
LBR	-1.97	-2.73	-1.72	-1.59
LCN2	2.57	2.14	4.63	-2.16
LDB1	-2.54	-2.93	-2.18	-1.35
LDHA	1.80	1.16	2.73	-2.36
LDHB	-2.94	-4.67	-5.33	1.14
LEF1	-1.98	-2.67	-2.57	-1.04
LEPROTL1	-2.62	-2.54	-3.30	1.30
LETM2	-6.36	-5.07	-5.88	1.16
LGALS1	1.58	1.17	2.87	-2.46
LGALS8	2.73	2.01	1.54	1.31
LILRA1	-4.68	-3.75	-5.34	1.43
LILRA2	-2.12	-2.37	-2.31	-1.03
LILRA5	2.08	1.43	1.88	-1.32
LIMK2	2.01	1.99	1.62	1.23
LIMS1	7.54	6.80	3.21	2.11
LIN7A	-1.52	-2.43	-1.71	-1.42
LIX1L	-1.88	-2.26	-1.86	-1.21
LMBRD1	-1.38	-1.91	-2.64	1.38
LMNB1	3.03	3.06	1.39	2.19
LOC152217	-2.03	-1.78	-1.97	1.11
LOC283588	-2.25	-2.16	-2.12	-1.02
LOC284757	2.24	1.04	2.17	-2.09
LOC285696	2.42	1.72	1.73	-1.00
LOC554203	-2.08	-2.07	-2.31	1.11
LOXHD1	2.62	1.79	2.25	-1.26

LPCAT1	1.74	2.01	1.18	1.70
LPCAT3	1.24	-1.55	1.38	-2.15
LPIN2	-1.86	-1.60	-2.58	1.61
LPPR2	-1.69	-2.22	-1.28	-1.73
LPXN	-3.52	-3.10	-3.88	1.25
LRMP	-3.93	-3.44	-5.90	1.71
LRRC25	-3.24	-2.60	-2.89	1.11
LRRC4	-2.58	-2.97	-2.15	-1.38
LRRC6	-1.27	-1.44	-2.51	1.74
LRRC8B	-1.29	-1.22	-2.17	1.78
LRRC8C	-2.00	-2.08	-2.14	1.03
LRRC8D	1.10	1.72	-1.56	2.69
LRRFIP1	-2.89	-3.24	-3.73	1.15
LRRN1	1.03	-1.83	1.50	-2.74
LSM10	-1.93	-1.53	-2.13	1.39
LSM14B	-2.54	-3.07	-1.82	-1.69
LSS	1.44	2.15	1.20	1.79
LTF	1.23	1.21	2.68	-2.23
LUC7L3	-1.86	-1.97	-2.43	1.23
LXN	-3.19	-3.22	-3.24	1.01
LY75	-2.03	-2.02	-3.81	1.89
LY96	1.26	1.05	-1.94	2.03
LYPLAL1	-1.84	-1.68	-2.16	1.29
LYRM5	-1.34	-1.95	-3.35	1.72
LYSMD3	-1.17	-1.19	-2.21	1.87
LZIC	-2.04	-2.25	-2.00	-1.13
M6PR	-2.41	-1.54	-2.39	1.55
MAD2L2	2.08	1.82	1.71	1.06
MAK	1.00	-1.19	-2.31	1.93
MALT1	-1.64	-2.09	-1.71	-1.22
MAML2	-1.63	1.31	-4.33	5.65
MAN1A1	-1.25	1.07	-2.72	2.92
MAN2A1	-1.95	-2.16	-2.96	1.37
MANBA	-1.37	-1.31	-2.35	1.80
MANBAL	-1.64	-1.38	-2.14	1.56
MANSC1	-1.78	-3.70	-1.41	-2.61
MAP1LC3A	1.88	2.43	1.17	2.07
MAP2K4	-1.84	-1.96	-3.17	1.62
MAP3K2	-1.95	-2.39	-3.20	1.34
MAP3K3	-2.52	-4.05	-2.00	-2.03
MAP3K5	-1.28	-1.31	-2.88	2.20
MAP3K7IP2	-1.02	1.15	-1.92	2.21
MAP3K8	1.27	1.74	-1.44	2.51
MAPK1	-1.60	-2.13	-1.53	-1.39
MAPK14	2.12	1.55	1.50	1.04
MAPK3	-1.60	-2.28	-1.53	-1.49
MAPK9	-1.16	-1.18	-2.25	1.91
MAPKAPK3	-2.19	-2.03	-2.25	1.11
MAPKSP1	-1.02	1.29	-1.56	2.02
MAPRE2	-1.70	-2.29	-2.30	1.01

MAPRE3	-2.81	-2.77	-3.30	1.19
MARCKS	2.70	2.89	1.67	1.74
MASTL	1.37	1.47	-1.36	2.00
MAT2A	-1.83	-2.06	-1.82	-1.13
MAT2B	1.01	1.11	-1.82	2.02
MATR3	-1.93	-1.93	-2.42	1.26
MBD4	-2.84	-3.95	-3.24	-1.22
MBIP	-2.74	-3.28	-2.54	-1.29
MBP	-2.85	-2.33	-2.64	1.13
MBTD1	-1.88	-2.14	-2.17	1.02
MCTP1	4.32	4.81	2.26	2.13
MDH2	-1.97	-2.30	-1.67	-1.37
MDM2	-1.27	1.05	-3.10	3.25
MDM4	-1.27	-1.59	-2.18	1.37
MDS2	-2.22	-2.45	-2.66	1.09
ME2	-1.71	-2.55	-1.37	-1.86
MEA1	-2.22	-2.29	-2.18	-1.05
MED25	-2.43	-2.51	-2.56	1.02
MEF2A	1.68	-1.36	1.82	-2.47
MEF2C	-4.06	-3.83	-4.17	1.09
METAP1	-2.24	-2.53	-2.30	-1.10
METTL4	-1.76	-1.67	-2.17	1.30
METTL9	1.96	1.35	2.01	-1.48
MFN1	-1.48	-1.80	-2.25	1.25
MFNG	-3.03	-3.12	-3.00	-1.04
MFSD2	1.37	2.82	1.15	2.44
MFSD5	-2.52	-2.19	-1.95	-1.12
MFSD8	-2.06	-1.82	-2.30	1.27
MGAT4A	-1.60	-3.07	-2.44	-1.26
MGAT5	-1.75	-1.48	-2.29	1.54
MGC16384	-2.49	-1.50	-4.20	2.81
MGC39372	1.31	2.39	1.13	2.12
MICAL2	-2.80	-4.05	-2.91	-1.39
MICALCL	-1.99	-2.05	-2.04	-1.01
MID1IP1	-3.72	-3.33	-4.16	1.25
MITD1	1.34	1.34	-1.59	2.13
MKLN1	-1.99	-2.66	-3.40	1.27
MKRN2	-1.98	-2.09	-1.77	-1.18
MLH1	2.96	3.18	1.63	1.95
MLLT6	2.68	2.64	1.87	1.41
MME	-1.15	-1.36	-3.09	2.26
MMP8	2.49	1.55	12.38	-7.96
MMP9	1.78	1.15	2.44	-2.12
MON2	-2.03	-2.45	-1.52	-1.62
MOP-1	-1.75	-2.12	-1.35	-1.57
MOSC1	-1.43	-2.25	-1.30	-1.73
MOSPD2	-1.45	-1.68	-2.35	1.40
MOV10	1.04	1.58	-1.54	2.42
MPHOSPH8	-1.60	-1.80	-2.12	1.17
MPPE1	-4.97	-5.00	-5.68	1.14

MPZL1	-4.80	-3.25	-7.88	2.43
MR1	2.22	2.18	1.23	1.78
MRPL51	-1.33	-1.77	1.30	-2.29
MRPS14	-2.53	-2.34	-2.04	-1.15
MS4A6A	1.22	-1.62	1.33	-2.16
MSL2	-1.54	-1.49	-2.00	1.34
MTFMT	-2.71	-2.63	-3.65	1.39
MTIF3	-1.68	-2.17	-1.45	-1.50
MTM1	-2.37	-3.27	-2.56	-1.28
MTMR10	-1.75	-2.29	-2.01	-1.14
MTMR12	-2.95	-3.44	-2.92	-1.18
MTRR	2.34	1.08	2.49	-2.30
MX1	2.24	2.50	1.19	2.09
MXI1	-2.18	-2.14	-2.05	-1.04
MYLIP	-1.63	-1.51	-2.62	1.73
MYO5A	-1.38	-2.31	-1.79	-1.29
MYST1	-1.96	-1.65	-2.08	1.26
MYST2	-1.52	-1.61	-2.01	1.25
MYT1L	-2.27	-2.24	-2.04	-1.10
N4BP1	2.09	2.07	1.31	1.58
NAAA	-4.58	-2.24	-4.60	2.05
NAB1	1.22	1.44	-2.11	3.04
NAPG	-2.46	-2.46	-2.36	-1.04
NARS	-1.98	-2.03	-1.43	-1.42
NAT13	-3.06	-2.70	-2.95	1.09
NBEAL1	-2.11	-2.37	-2.46	1.04
NBN	3.89	3.87	2.02	1.92
NCAPD2	-1.29	-1.74	1.17	-2.03
NCK1	-1.88	-1.96	-2.44	1.24
NCKAP1L	-1.56	-2.69	-1.13	-2.37
NCL	-2.04	-1.80	-2.48	1.38
NCOA1	-1.35	-1.71	-2.16	1.27
NCOA3	-1.33	-2.16	-1.34	-1.61
NCOA4	-1.31	-1.09	-2.17	1.98
NCOA6	-1.64	-2.34	-1.54	-1.52
NCOR1	-1.41	-2.23	-1.44	-1.56
NCRNA00152	2.37	2.36	1.09	2.16
NCRNA00173	-2.52	-2.64	-3.71	1.41
NCRNA00189	1.72	1.77	-1.66	2.94
NCRNA00204	-1.39	-1.54	-4.78	3.10
NDE1	-1.76	-1.50	-2.69	1.79
NDRG3	-1.33	-2.10	-1.83	-1.15
NDST1	-1.75	-2.39	-1.67	-1.43
NDST2	3.13	1.84	3.16	-1.72
NDUFB3	1.81	1.88	-1.14	2.14
NDUFB5	-2.10	-2.02	-2.23	1.11
NDUFB6	-1.45	-1.48	-2.75	1.86
NDUFS3	-1.83	-2.02	-1.51	-1.34
NEDD4	2.32	1.21	2.41	-2.00
NEDD9	-1.59	1.04	-2.93	3.05

NEK6	-2.82	-3.94	-2.38	-1.66
NEK7	1.04	-1.30	-2.26	1.74
NFATC3	-2.23	-2.85	-2.29	-1.24
NFE2L1	-2.09	-2.29	-1.75	-1.31
NFE2L2	1.41	1.62	-1.60	2.59
NF-E4	-1.50	-1.39	-3.36	2.42
NFKB1	3.27	3.65	1.59	2.29
NFKB2	3.13	3.39	2.32	1.46
NFKBIE	1.76	2.51	1.12	2.24
NFKBIZ	2.00	2.03	1.17	1.74
NFXL1	-3.15	-2.31	-3.83	1.66
NGLY1	-1.77	-2.56	-2.40	-1.06
NIACR1	2.67	3.02	1.39	2.17
NIACR2	2.91	3.19	1.36	2.35
NIN	-1.35	-2.45	-1.31	-1.87
NINJ1	-1.04	1.14	-1.83	2.09
NKTR	-1.96	-2.57	-2.38	-1.08
NLRC4	2.59	2.00	1.68	1.19
NLRP1	-1.41	-1.43	-2.08	1.46
NLRP3	3.55	4.06	1.25	3.25
NLRP6	-2.54	-1.40	-2.93	2.09
NMI	1.02	1.12	-2.58	2.90
NONO	-1.97	-1.87	-2.31	1.23
NOV	-2.76	-2.53	-3.45	1.36
NPC2	-1.82	-1.36	-2.94	2.16
NPL	-1.27	-1.37	-2.94	2.14
NPM1	-1.46	-2.24	-2.01	-1.11
NR3C1	-1.65	-1.94	-2.09	1.08
NSD1	-2.36	-2.71	-2.23	-1.22
NSF	-2.34	-2.85	-1.80	-1.58
NSUN6	2.35	2.04	1.37	1.48
NSUN7	2.09	1.37	1.16	1.18
NUB1	1.20	1.41	-2.29	3.23
NUBP1	1.02	1.66	-1.34	2.22
NUDT21	-3.42	-4.21	-2.73	-1.54
NUDT3	-1.59	-1.69	-2.01	1.19
NUDT5	-1.52	-1.29	-2.42	1.88
NUP153	-1.92	-1.92	-2.91	1.52
NUP43	-1.98	-2.26	-2.01	-1.13
OAS1	1.46	1.73	-1.32	2.27
OAS2	1.76	2.19	-1.27	2.77
OAS3	2.67	3.17	1.41	2.24
OASL	-1.08	1.28	-1.94	2.48
OAZ2	-1.93	-1.51	-2.86	1.90
OCR1	-1.63	-2.32	-5.89	2.54
ODZ1	2.97	1.30	3.71	-2.86
OGFRL1	-1.89	-1.79	-2.92	1.63
OLAH	3.39	2.17	1.84	1.18
OLFM4	1.47	1.17	3.84	-3.28
OLR1	10.25	11.17	8.70	1.28

OR5AU1	2.10	1.32	1.04	1.27
ORAI3	-2.32	-1.90	-1.91	1.01
ORC4L	-1.42	-1.83	-2.35	1.28
ORM1	7.02	6.73	4.06	1.66
ORM2	6.66	7.15	3.35	2.14
ORMDL1	-2.03	-2.48	-2.15	-1.15
OSBPL11	-1.43	-1.49	-2.63	1.77
OSGIN2	-1.66	-1.56	-3.48	2.23
OSM	3.17	2.74	2.67	1.03
OSTalpha	2.11	1.50	2.14	-1.43
OSTM1	-1.42	-1.44	-2.11	1.47
OXA1L	-2.19	-2.21	-1.74	-1.27
OXR1	-1.84	-2.30	-2.51	1.09
P2RX1	-1.43	-2.13	-1.27	-1.68
P2RY10	-11.76	-7.18	-11.63	1.62
P2RY14	2.86	3.20	1.39	2.30
P4HA1	1.78	1.98	-1.07	2.12
PACS1	-1.58	-2.13	-1.94	-1.10
PADI4	1.63	-1.11	1.92	-2.12
PAG1	2.34	1.81	2.32	-1.28
PAK1	-1.47	-1.37	-2.04	1.49
PAK2	-1.90	-1.85	-2.41	1.30
PAPD5	-3.79	-4.04	-3.90	-1.04
PARN	-1.77	-2.07	-2.31	1.12
PARP12	1.42	1.64	-1.23	2.01
PARP14	1.75	2.22	-1.67	3.71
PARP16	-1.78	-2.21	-1.90	-1.17
PARP8	-1.40	-1.39	-2.88	2.07
PARP9	1.30	1.65	-1.54	2.54
PBX2	-1.36	-1.76	-2.84	1.61
PBXIP1	-2.17	-2.10	-2.33	1.11
PCGF5	-2.16	-1.83	-3.78	2.06
PCM1	-1.18	-2.33	-1.68	-1.39
PCMT1	-1.51	-2.29	1.04	-2.39
PCMTD1	-1.05	-1.33	-2.02	1.51
PCMTD2	-2.24	-2.69	-2.88	1.07
PCTK2	-1.56	-1.59	-2.06	1.30
PDCD1LG2	1.19	2.29	-1.08	2.47
PDCD4	-1.13	-1.01	-3.41	3.38
PDE3B	-1.15	-2.03	-1.62	-1.25
PDE4B	1.23	1.48	-2.03	3.01
PDE7A	-2.59	-3.03	-3.48	1.15
PDIA6	-1.86	-1.15	-2.62	2.27
PDLIM2	-2.42	-2.23	-2.32	1.04
PDLIM5	1.40	1.75	-1.15	2.01
PDPK1	-1.66	-2.26	-1.25	-1.80
PDS5B	-1.19	-2.07	-1.86	-1.11
PDSS1	2.72	1.78	2.44	-1.37
PDZD8	-1.91	-3.45	-1.25	-2.77
PECAM1	-4.68	-3.30	-5.57	1.69

PECR	2.05	1.02	2.46	-2.40
PELI1	1.48	1.51	-1.34	2.03
PELI2	-2.02	-2.42	-2.08	-1.16
PER3	-2.52	-2.15	-1.69	-1.27
PFKFB2	3.33	1.50	4.30	-2.87
PFKFB3	3.96	3.04	3.83	-1.26
PGCP	-1.28	-1.26	-2.15	1.71
PGLYRP1	1.35	-1.10	2.25	-2.49
PGM2	-2.71	-4.13	-2.05	-2.02
PGRMC2	-2.18	-2.21	-2.41	1.09
PHF11	1.44	1.33	-1.70	2.26
PHF17	-2.22	-2.15	-2.03	-1.06
PHF20	-1.49	-1.23	-2.33	1.90
PHF3	-1.61	-2.05	-2.43	1.19
PHKG2	-1.76	-2.15	-1.54	-1.40
PHOSPHO1	-8.71	-7.85	-10.27	1.31
PI3	-2.32	1.89	-2.93	5.53
PI4K2B	1.46	2.47	1.07	2.31
PICALM	-1.18	-1.34	-2.12	1.59
PIGB	-4.71	-3.94	-6.30	1.60
PIGS	-2.38	-2.80	-1.69	-1.66
PIGX	-4.51	-2.63	-10.23	3.88
PIH1D1	-1.86	-1.91	-3.03	1.58
PIK3AP1	2.69	3.02	2.01	1.50
PIK3C2A	-1.82	-2.06	-2.40	1.17
PIK3CA	-1.24	-1.50	-2.31	1.54
PIK3CB	-1.92	-2.55	-1.90	-1.34
PIK3R1	-2.65	-3.64	-1.91	-1.90
PIKFYVE	-1.76	-2.19	-2.16	-1.01
PIP4K2C	-2.55	-2.64	-2.19	-1.21
PIP5K1B	-1.74	-2.01	-1.70	-1.18
PISD	-2.35	-2.07	-2.35	1.13
PKM2	1.41	-1.13	2.47	-2.79
PKN2	-2.77	-2.98	-5.49	1.84
PLAGL2	2.65	3.18	1.31	2.44
PLAU	1.39	2.87	1.16	2.48
PLCG2	-1.80	-2.18	-1.94	-1.12
PLD1	1.91	2.38	-1.08	2.56
PLEKHA7	-1.70	-2.05	-2.13	1.04
PLEKHF2	1.75	2.06	-1.65	3.40
PLEKHO1	-2.60	-1.62	-4.68	2.89
PLIN5	2.81	2.92	1.94	1.50
PLK3	2.81	3.42	1.67	2.05
PLP2	-1.05	-1.66	1.29	-2.14
PLSCR1	4.27	3.73	2.69	1.38
PMAIP1	-4.37	-1.64	-6.00	3.65
PML	1.97	2.15	1.27	1.69
PMS2CL	-1.61	-1.45	-2.70	1.86
PNKD	1.57	2.07	1.05	1.97
PNPLA8	1.33	1.26	-1.71	2.15

PNPT1	1.72	2.44	-1.14	2.77
POLB	-1.36	-1.03	-3.18	3.09
POLR1D	-1.98	-2.22	-1.71	-1.30
POLR2B	-1.86	-1.93	-2.48	1.28
POLR2C	-1.64	-2.07	-1.68	-1.23
POLR2F	-1.92	-2.24	-1.83	-1.23
POLR2G	-2.38	-2.54	-2.12	-1.20
POLR3K	-2.48	-2.50	-2.40	-1.04
POR	2.38	1.73	2.11	-1.22
POU2F1	-1.72	-1.67	-2.06	1.23
PPCDC	-1.40	-1.02	-2.08	2.04
PPCS	-1.79	-2.04	-2.14	1.05
PPFIA1	-2.23	-2.50	-2.53	1.01
PPFIBP2	-1.65	-2.62	-1.14	-2.30
PPIB	-2.27	-1.86	-1.55	-1.20
PPIF	1.25	2.01	-2.36	4.73
PPM1F	-1.82	-1.37	-2.27	1.65
PPM1M	-1.64	-2.27	-1.42	-1.60
PPP1CA	-2.01	-3.07	-1.49	-2.06
PPP1CC	-5.75	-3.76	-8.28	2.20
PPP1R7	-2.04	-2.40	-2.00	-1.20
PPP2R1A	-1.62	-2.21	-1.24	-1.78
PPP2R2A	-1.21	-1.17	-2.34	1.99
PPP3CA	-1.30	-1.66	-2.11	1.27
PPP4R2	2.27	1.82	1.45	1.25
PPT1	-1.63	-1.44	-2.95	2.05
PPTC7	-2.98	-2.36	-5.22	2.21
PRAM1	-1.94	-2.37	-1.62	-1.46
PRDM1	2.18	2.59	-1.30	3.37
PRDM8	2.48	3.02	1.38	2.19
PRKAA1	-1.82	-2.12	-2.03	-1.04
PRKACB	-1.94	-2.33	-2.38	1.02
PRKAG2	-1.87	-1.48	-3.23	2.18
PRMT5	-1.28	-1.83	-2.02	1.11
PRPF18	-1.37	-2.04	-1.61	-1.27
PRPF40A	-1.56	-1.53	-2.05	1.34
PRPF4B	-1.84	-1.62	-2.29	1.42
PRPF8	-1.70	-2.02	-1.97	-1.03
PRPSAP1	1.99	2.34	1.28	1.82
PRR5L	2.10	2.94	1.13	2.60
PRRG4	-1.98	-1.35	-5.31	3.94
PRUNE	-2.43	-2.59	-2.61	1.01
PSD4	-1.99	-1.87	-2.40	1.29
PSIP1	-2.94	-3.17	-3.07	-1.03
PSMA3	1.34	1.60	-1.68	2.68
PSMB1	-1.28	-1.05	-2.28	2.18
PSMB2	-2.27	-2.18	-2.06	-1.06
PSMB8	1.17	1.31	-1.73	2.27
PSMB9	-1.17	1.08	-1.89	2.03
PSMD5	-1.50	-2.21	-1.34	-1.64

PSME3	-1.97	-2.06	-1.91	-1.08
PSME4	-1.42	-1.68	-2.39	1.42
PSMF1	-1.70	-2.07	-1.38	-1.50
PSTPIP1	-2.02	-2.33	-1.71	-1.36
PSTPIP2	6.41	6.02	4.98	1.21
PTBP1	-2.04	-1.92	-1.99	1.04
PTDSS1	-2.17	-2.24	-1.67	-1.34
PTGER2	2.65	2.80	1.83	1.54
PTGER4	-3.83	-1.63	-5.33	3.27
PTP4A2	-1.85	-2.14	-2.07	-1.03
PTP4A3	-3.33	-1.47	-3.99	2.72
PTPN12	1.34	1.39	-1.57	2.17
PTPN2	3.00	2.14	1.60	1.33
PTRH2	-2.06	-1.52	-3.61	2.37
PTX3	2.47	1.77	1.65	1.07
PUM2	-1.41	-1.48	-2.45	1.65
PUS3	2.22	1.70	2.17	-1.27
PYCARD	-2.33	-2.45	-2.15	-1.14
PYGL	1.03	-1.62	1.24	-2.02
QARS	-1.70	-2.15	-1.39	-1.55
QKI	1.29	1.54	-1.83	2.83
QRICH1	-1.77	-2.19	-1.69	-1.30
QSK	1.79	2.07	-1.16	2.39
RAB11FIP1	-3.26	-3.63	-3.03	-1.20
RAB11FIP2	-1.63	-2.54	-1.38	-1.84
RAB11FIP4	-2.28	-2.09	-1.85	-1.13
RAB18	-1.67	-2.27	-2.06	-1.10
RAB1A	1.46	1.37	-1.59	2.18
RAB20	2.76	3.56	1.65	2.16
RAB21	1.69	1.91	-1.09	2.08
RAB2A	-1.38	-1.44	-2.66	1.85
RAB37	-3.87	-4.79	-3.88	-1.23
RAB7L1	3.98	2.24	2.57	-1.14
RAB8A	-1.60	-1.41	-2.00	1.42
RABEP1	-1.76	-1.65	-2.08	1.26
RABGEF1	3.24	2.25	2.63	-1.17
RABL2B	-2.06	-1.97	-1.76	-1.12
RAC1	-1.57	-1.35	-2.31	1.71
RAF1	-1.76	-1.71	-2.56	1.50
RALGDS	4.24	5.91	1.59	3.71
RALGPS2	-1.20	-2.10	-1.62	-1.29
RANBP2	3.18	3.20	1.13	2.83
RAP1A	-1.53	-1.38	-2.38	1.73
RAP1B	2.45	1.75	1.04	1.69
RAPGEF2	1.37	1.35	-1.75	2.36
RASA1	-1.54	-2.07	-1.88	-1.10
RASA3	-2.14	-2.17	-2.30	1.06
RASGRP2	-1.65	-2.09	-1.50	-1.39
RASSF3	-1.69	-1.84	-2.64	1.44
RBBP5	-1.85	-1.60	-2.08	1.30

RBBP7	-2.08	-1.97	-2.03	1.03
RBM15	-1.79	-2.21	-1.72	-1.29
RBM18	-1.56	-1.46	-2.04	1.40
RBM25	-1.30	-1.39	-2.50	1.80
RBM26	-2.02	-2.36	-2.13	-1.11
RBM43	1.13	2.02	-1.36	2.75
RBM4B	-2.86	-2.65	-2.66	1.00
RCBTB2	-3.39	-3.45	-4.31	1.25
RCHY1	2.36	1.52	1.68	-1.11
RCOR1	-1.97	-2.56	-2.15	-1.19
RCOR3	-1.47	-2.04	-1.81	-1.12
RCSD1	-2.29	-1.80	-1.92	1.07
REEP3	-1.71	-2.42	-2.10	-1.15
RELA	1.78	2.24	1.26	1.78
RELB	2.31	2.21	1.63	1.36
RELL1	-2.71	-2.18	-3.51	1.61
REM2	-1.94	-1.81	-2.67	1.47
REST	-1.81	-2.06	-1.86	-1.11
REV3L	-1.77	-1.64	-2.82	1.73
RFWD2	-1.24	-1.74	-3.12	1.79
RFX5	1.97	2.18	1.30	1.67
RGS14	-2.23	-2.56	-1.63	-1.57
RHBDF2	2.00	2.35	1.35	1.75
RHOA	1.32	1.26	-1.61	2.03
RHOH	7.06	9.92	2.84	3.50
RICTOR	-1.27	-1.12	-3.26	2.92
RIF1	1.55	1.37	-1.61	2.21
RIPK1	1.20	1.20	-1.97	2.37
RIPK2	2.64	3.09	-1.47	4.56
RIT1	-1.24	-1.01	-2.19	2.18
RLF	1.17	1.36	-1.73	2.35
RMI1	-2.33	-2.48	-2.70	1.09
RNASE6	-2.90	-2.52	-3.44	1.37
RNASET2	-2.17	-1.94	-3.71	1.91
RNF103	-2.07	-1.79	-3.00	1.68
RNF114	-1.91	-1.58	-2.07	1.31
RNF122	-1.59	-2.00	-2.28	1.14
RNF125	-3.01	-3.21	-3.31	1.03
RNF130	-1.46	-1.40	-2.28	1.63
RNF135	-1.79	-2.03	-1.96	-1.04
RNF14	-2.33	-2.00	-2.32	1.16
RNF141	-1.66	-1.63	-2.24	1.38
RNF144B	1.92	2.22	-1.03	2.28
RNF160	-1.51	-1.57	-2.05	1.31
RNF166	-2.68	-2.74	-2.52	-1.09
RNF19B	1.10	1.33	-1.81	2.41
RNF20	-1.87	-1.89	-2.15	1.14
RNF38	-1.51	-2.17	-1.51	-1.44
RNF44	-1.60	-2.07	-1.54	-1.34
RNPEP	-1.83	-1.93	-2.02	1.05

RNPS1	-2.20	-1.87	-1.38	-1.35
ROCK2	-1.44	-2.02	-1.92	-1.05
ROPN1L	-1.03	-1.80	1.12	-2.02
RP6-213H19.1	1.08	1.55	-1.48	2.30
RPAP3	-5.59	-4.26	-6.67	1.57
RPGR	1.01	1.07	-2.18	2.33
RPL15	-2.39	-2.46	-2.88	1.17
RPL17	-1.61	-1.61	-2.11	1.32
RPL23AP32	-1.93	-1.40	-2.51	1.79
RPL23AP64	-1.64	-1.70	-2.66	1.57
RPL28	-1.10	-1.30	-2.22	1.72
RPL35	-2.15	-2.11	-1.98	-1.06
RPL41	-1.74	-1.69	-2.17	1.28
RPL6	-2.00	-2.33	-1.73	-1.34
RPL9	-1.83	-1.84	-2.41	1.31
RPN1	-2.47	-1.95	-3.10	1.59
RPP38	-2.35	-1.87	-2.25	1.20
RPRD1A	-2.52	-2.60	-2.78	1.07
RPS6KA2	1.33	1.06	2.11	-1.99
RPS6KA3	-1.16	-1.67	-2.10	1.26
RPS6KA5	-5.09	-5.07	-7.33	1.45
RRAGC	-1.85	-1.47	-2.10	1.43
RRM2B	-1.56	-1.84	-2.52	1.37
RRP12	-2.56	-2.70	-2.18	-1.24
RSAD2	1.18	1.95	-2.04	3.97
RSBN1	2.23	1.40	1.95	-1.39
RSBN1L	-2.89	-2.90	-4.14	1.43
RSL1D1	-1.90	-1.99	-2.27	1.14
RTCD1	-1.98	-2.02	-1.77	-1.14
RTF1	-2.13	-3.17	-1.84	-1.72
RUFY1	-1.46	1.01	-1.99	2.01
RUNDC1	-1.14	-1.73	1.52	-2.63
RUNX2	-3.38	-3.99	-4.02	1.01
RWDD4A	-2.22	-2.24	-1.59	-1.40
RYBP	-1.55	-1.56	-2.24	1.44
S100A10	-4.42	-4.91	-7.24	1.48
S1PR1	2.31	1.25	2.73	-2.19
SACM1L	-2.45	-3.15	-2.48	-1.27
SAMD9	1.37	1.46	-1.75	2.55
SAMD9L	1.67	1.76	-1.30	2.28
SAMHD1	2.76	3.00	1.03	2.92
SAMSN1	8.97	7.97	5.58	1.43
SAP130	-2.40	-2.99	-2.33	-1.28
SAPS1	-1.66	-2.09	-1.44	-1.45
SAR1B	-1.80	-1.37	-2.24	1.64
SARS	-1.84	-2.34	-1.83	-1.28
SART1	2.10	1.27	-2.12	2.69
SART3	-2.09	-1.42	-1.48	1.04
SASS6	-2.08	-2.10	-2.29	1.09
SBF2	-1.08	-1.63	-2.02	1.24

SC4MOL	2.04	1.20	1.91	-1.59
SCAMP1	-3.06	-3.63	-3.20	-1.13
SCAP	-2.41	-2.19	-2.36	1.08
SCLT1	-1.83	-1.78	-3.91	2.19
SCRN3	-2.27	-1.79	-3.14	1.76
SDC2	-2.26	-1.97	-2.18	1.10
SDHA	-1.38	-2.02	-1.35	-1.50
SDHB	-1.60	-1.86	-2.41	1.30
SEC14L1	-1.65	-1.44	-2.21	1.54
SEC22B	2.45	2.47	1.14	2.18
SEC23A	-1.53	-2.41	-1.26	-1.91
SEC24A	1.31	1.47	-1.44	2.12
SEC24D	-1.71	-1.49	-3.22	2.17
SEC61A2	-1.68	-1.51	-2.34	1.55
SECISBP2	-1.28	-1.09	-2.31	2.11
SELPLG	-1.69	-2.18	-1.42	-1.53
SEMA4D	-2.16	-2.14	-2.24	1.05
SENP6	-2.04	-2.33	-3.03	1.30
SENP7	-1.21	-2.07	-2.09	1.01
SERBP1	-2.90	-2.82	-3.80	1.35
SERPINB1	2.03	1.92	1.44	1.34
SERPINB10	1.19	1.19	2.84	-2.39
SERPINB9	3.83	6.78	1.48	4.58
SERPING1	-1.13	1.17	-2.74	3.21
SERTAD2	2.30	2.85	-1.10	3.14
SESN3	-3.63	-3.99	-3.66	-1.09
SESTD1	1.07	1.49	-1.59	2.37
SET	-4.31	-3.65	-5.11	1.40
SETD2	-1.60	-1.98	-2.08	1.05
SETD3	-1.73	-2.16	-1.80	-1.20
SETX	-1.66	-1.71	-2.81	1.64
SFRS2IP	-1.39	-1.40	-2.46	1.76
SFRS3	1.07	1.01	-2.10	2.13
SFRS7	-1.35	-1.16	-2.17	1.87
SFXN1	1.48	-1.01	2.00	-2.02
SGK1	-1.40	1.12	-2.66	2.97
SGK196	-2.00	-2.03	-2.59	1.28
SGPP2	11.43	14.14	4.80	2.95
SH2B3	-2.12	-1.26	-2.10	1.67
SH2D3A	3.11	3.09	2.16	1.43
SH3BP5	2.78	1.92	1.90	1.01
SIGLEC10	-1.76	1.16	-2.91	3.39
SIPA1L2	2.30	1.00	2.00	-1.99
SIRPB2	-2.10	-2.87	-2.51	-1.14
SIRPD	-1.68	-2.08	-1.71	-1.21
SIRT1	-2.10	-1.60	-2.74	1.71
SLAMF1	2.09	1.99	1.38	1.43
SLAMF6	-3.48	-2.02	-3.96	1.96
SLAMF7	12.87	18.33	3.44	5.33
SLAMF8	1.76	3.31	1.30	2.55

SLBP	-5.43	-5.96	-6.15	1.03
SLC11A2	6.76	7.74	2.66	2.91
SLC15A4	-3.48	-1.49	-5.33	3.57
SLC16A5	-2.18	-2.02	-2.05	1.02
SLC16A6	-2.93	-2.43	-3.30	1.36
SLC17A5	-1.94	-2.19	-1.97	-1.11
SLC1A3	8.62	3.92	5.31	-1.35
SLC20A1	1.66	2.17	-1.04	2.26
SLC22A15	-1.40	-2.38	-1.39	-1.71
SLC23A2	-1.96	-2.30	-2.26	-1.02
SLC25A1	-1.86	-2.05	-1.79	-1.14
SLC25A13	-1.34	1.50	-1.52	2.28
SLC25A20	-1.83	-2.18	-1.34	-1.63
SLC25A3	-1.89	-2.04	-1.76	-1.16
SLC25A40	2.43	1.06	2.32	-2.19
SLC25A46	-1.80	-2.18	-1.80	-1.21
SLC29A1	1.46	2.15	1.04	2.08
SLC30A7	2.71	1.74	2.04	-1.17
SLC31A1	-2.99	-2.54	-2.83	1.11
SLC31A2	-1.41	-1.13	-2.32	2.06
SLC35B2	1.74	2.26	1.20	1.88
SLC35E1	-2.64	-2.68	-2.26	-1.18
SLC35E3	-1.75	-1.06	-2.76	2.60
SLC36A1	-1.95	-2.05	-1.81	-1.13
SLC36A4	2.41	1.64	2.22	-1.35
SLC37A3	2.47	1.32	2.23	-1.69
SLC38A1	-1.94	1.09	-2.79	3.03
SLC39A6	-2.38	-2.06	-2.78	1.35
SLC39A8	5.34	4.40	4.61	-1.05
SLC40A1	-2.99	-4.96	-2.30	-2.16
SLC43A3	3.11	5.75	1.65	3.49
SLC44A2	-1.73	-2.07	-1.74	-1.19
SLC5A3	-2.12	-1.62	-2.03	1.25
SLC7A5	2.59	2.79	1.59	1.75
SLC7A6	-2.29	-2.52	-2.43	-1.04
SLC7A7	-3.64	-1.75	-3.31	1.89
SLC8A1	-2.21	-2.09	-3.77	1.80
SLC9A3R1	-2.49	-3.00	-1.96	-1.53
SLC9A6	-1.40	-2.24	1.05	-2.35
SLCO4C1	-1.33	-2.24	-1.11	-2.02
SLFN5	-1.01	1.24	-1.99	2.48
SLMAP	-1.44	-2.51	-1.90	-1.32
SLMO2	-1.32	-1.13	-2.09	1.84
SLPI	-2.13	1.88	-4.50	8.46
SLTM	-2.14	-2.79	-1.88	-1.49
SLU7	1.04	1.08	-2.35	2.54
SMAD2	-2.04	-2.75	-2.01	-1.37
SMAD3	2.06	2.28	1.51	1.50
SMARCA2	-2.04	-2.97	-2.35	-1.27
SMARCA5	-1.70	-1.62	-2.57	1.59

SMARCC2	-2.18	-2.74	-2.12	-1.29
SMC3	-3.15	-2.91	-2.99	1.03
SMCHD1	-1.93	-1.75	-5.46	3.13
SMG7	1.26	1.55	-1.50	2.33
SMNDC1	-1.32	-1.29	-2.06	1.60
SMPDL3A	2.11	1.35	2.17	-1.61
SMURF2	-1.74	-2.25	-2.18	-1.03
SNAP29	-1.90	-2.37	-1.78	-1.34
SNAPC5	-2.56	-5.53	-2.91	-1.90
SNORD49A	2.08	1.23	2.18	-1.78
SNRK	-1.76	-1.64	-2.27	1.38
SNRNP200	-1.44	-1.58	-2.01	1.28
SNRNP48	-2.46	-3.20	-3.05	-1.05
SNTB2	-1.50	-2.03	-1.24	-1.64
SNX10	1.25	1.43	-1.62	2.31
SNX13	-1.14	-1.36	-2.11	1.55
SNX2	-2.18	-2.12	-3.81	1.79
SNX3	1.76	-1.08	2.80	-3.03
SOCS3	4.60	5.05	3.14	1.61
SORT1	2.36	1.65	1.90	-1.16
SP140	1.47	1.67	-1.45	2.42
SP140L	1.20	1.46	-1.42	2.07
SPAG7	1.11	1.44	-1.44	2.07
SPAG9	-1.46	-1.03	-2.89	2.80
SPAST	-1.94	-2.17	-2.86	1.31
SPATA6	-1.91	-1.55	-2.24	1.44
SPATC1	2.01	1.82	1.66	1.10
SPCS3	-1.55	-1.91	-2.18	1.14
SPEN	-2.72	-2.19	-2.57	1.17
SPINT2	2.50	2.69	2.21	1.22
SPOP	-1.67	-1.61	-2.18	1.36
SPOPL	-1.44	-2.07	-2.55	1.23
SPP1	3.10	1.62	1.23	1.32
SPRED2	2.06	1.56	-1.00	1.56
SPRYD3	-2.84	-2.64	-2.21	-1.20
SPTLC2	-1.03	-2.05	1.15	-2.34
SQSTM1	1.23	1.73	-1.30	2.25
SRP14	-1.16	-1.63	-3.44	2.11
SRP72	-2.03	-1.74	-2.03	1.17
SRPK2	-2.10	-2.88	-3.66	1.27
SRXN1	-2.96	-1.71	-4.48	2.62
SSBP2	-1.35	-1.68	-2.40	1.43
SSBP4	-1.73	-1.79	-2.04	1.14
SSNA1	-2.25	-2.34	-2.02	-1.16
SSR3	-2.22	-2.31	-1.98	-1.17
ST3GAL4	2.16	1.70	1.39	1.22
ST3GAL6	-3.72	-2.72	-4.63	1.70
ST6GAL1	-2.75	-3.13	-2.67	-1.17
ST8SIA4	-1.31	-1.46	-3.50	2.40
STAG1	-1.19	-1.60	-2.26	1.41

STAM	-1.78	-2.29	-1.48	-1.55
STAM2	-1.09	-1.14	-2.09	1.84
STAMBP	-1.56	-1.47	-2.12	1.44
STAP1	-1.87	-1.68	-2.58	1.54
STARD3NL	-1.38	1.78	-1.85	3.29
STARD7	-2.74	-1.97	-2.06	1.05
STAT1	1.21	1.45	-1.62	2.34
STAT2	1.58	2.11	-1.31	2.76
STAT4	2.25	2.17	1.11	1.96
STAT5A	1.75	1.95	-1.03	2.00
STIM2	-1.90	-2.17	-1.98	-1.10
STIP1	1.21	1.88	-1.15	2.16
STK10	-1.57	-1.60	-2.03	1.27
STK38L	-1.87	-2.36	-2.07	-1.14
STK40	-1.40	-1.13	-2.06	1.84
STOM	5.86	4.00	6.09	-1.52
STRN4	-2.37	-2.14	-2.24	1.04
STT3A	-2.01	-1.29	-1.62	1.25
STT3B	-1.93	-2.29	-2.35	1.02
STX10	-2.31	-2.88	-2.17	-1.33
STX6	-1.74	-2.02	-2.25	1.12
STX7	-1.38	-1.85	-2.40	1.30
STXBP3	-1.01	-1.27	-2.54	2.00
STYX	-3.09	-4.06	-2.67	-1.52
SULF2	-1.83	-2.50	-3.23	1.29
SULT1A2	-1.37	-2.07	-1.16	-1.79
SULT1B1	-1.23	-2.88	-1.27	-2.27
SUMO1P1	-1.52	-1.57	-2.14	1.36
SUMO2	-1.86	-2.41	-2.40	-1.00
SUPT16H	-2.47	-2.48	-2.72	1.10
SUSD3	1.48	1.17	2.27	-1.94
SUZ12	-1.90	-2.15	-2.53	1.18
SYF2	-1.15	-1.16	-2.35	2.02
SYK	-2.20	-2.09	-2.13	1.02
SYNE1	3.29	1.57	3.72	-2.37
TACC1	-2.57	-2.10	-2.97	1.42
TACC3	-1.90	-2.41	-2.45	1.02
TAF12	-1.61	-1.67	-2.09	1.25
TAF1D	-1.39	-1.64	-2.40	1.46
TAGAP	-1.18	-1.03	-2.03	1.97
TAGLN2	-1.90	-1.61	-2.20	1.36
TANK	1.55	2.06	-1.74	3.59
TAOK3	-1.72	-2.03	-2.27	1.12
TAP2	-1.13	1.16	-1.98	2.29
TAPBPL	-2.16	-1.62	-2.84	1.75
TAPT1	-2.04	-2.14	-1.96	-1.09
TARDBP	-1.36	-2.02	-2.18	1.08
TAX1BP3	-1.89	-1.78	-2.40	1.35
TBC1D1	-1.92	-2.45	-2.78	1.13
TBC1D15	2.24	1.70	1.18	1.44

TBC1D23	-1.02	1.08	-1.87	2.01
TBC1D8	4.80	2.35	5.47	-2.33
TBK1	1.41	1.77	-1.50	2.65
TBL1XR1	-1.24	-1.46	-2.25	1.54
TBP	-2.13	-2.03	-2.08	1.03
TC2N	-2.01	-2.30	-2.28	-1.01
TCEB3	-2.05	-1.54	-1.73	1.12
TCF7L2	2.43	2.63	1.65	1.59
TCFL5	1.00	2.45	-1.78	4.37
TCN1	1.81	1.08	2.41	-2.22
TCP11L2	-1.72	-2.32	-1.97	-1.18
TCTN1	-2.50	-2.14	-2.45	1.15
TESK2	1.67	1.69	-1.27	2.15
TFDP1	2.34	1.27	2.85	-2.24
TFDP2	3.11	1.33	3.42	-2.58
TFEB	-2.04	-2.03	-2.18	1.07
TFEC	5.63	13.99	1.82	7.68
TFRC	6.88	7.00	3.02	2.32
TGFBR1	-2.33	-3.17	-1.54	-2.07
TGIF2	1.67	2.05	1.03	2.00
THAP5	1.04	1.08	-1.89	2.03
THAP6	-1.58	-1.53	-2.35	1.53
THBD	-2.23	-1.62	-3.34	2.05
THOC7	1.14	-1.07	-2.16	2.02
TICAM2	-1.96	-1.77	-2.33	1.32
TIFA	8.76	8.80	3.03	2.91
TIGD1	-2.02	-1.30	-2.07	1.60
TIGD3	-3.60	-3.19	-2.64	-1.21
TIMM23	-2.10	-2.14	-1.86	-1.15
TIMM8B	-2.32	-1.95	-2.02	1.04
TIMP1	-1.14	1.03	-2.00	2.06
TIMP2	-1.82	-2.43	-1.56	-1.56
TKT	-1.57	-2.56	-1.47	-1.75
TLR1	1.17	1.16	-1.88	2.19
TLR10	-1.54	-1.35	-2.40	1.78
TLR2	1.38	1.70	-1.77	3.00
TLR5	3.39	2.73	1.88	1.45
TLR6	-1.34	-1.56	-2.62	1.68
TMCC1	-1.94	-1.11	-4.83	4.36
TMCC3	-1.81	-2.04	-1.71	-1.19
TMED10	-2.10	-1.48	-1.76	1.19
TMED2	-1.41	-1.54	-2.02	1.31
TMED8	2.38	1.20	2.42	-2.02
TMEM111	-1.43	-1.46	-2.05	1.40
TMEM120B	-3.26	-2.24	-2.74	1.22
TMEM140	1.23	1.41	-1.57	2.22
TMEM14B	-2.31	-2.41	-1.85	-1.30
TMEM164	-1.84	-1.97	-2.54	1.29
TMEM165	2.45	2.44	1.20	2.04
TMEM185B	2.30	2.60	1.89	1.37

TMEM188	-1.35	-1.41	-2.05	1.45
TMEM205	2.18	2.31	1.79	1.29
TMEM216	-2.37	-2.39	-1.73	-1.38
TMEM222	-2.28	-1.90	-2.07	1.09
TMEM45B	-1.95	-2.34	-1.90	-1.23
TMEM55A	-2.21	-2.88	-2.43	-1.18
TMEM65	-1.69	-2.35	-1.68	-1.40
TMEM66	-1.66	-1.63	-2.04	1.25
TMEM9B	-1.89	-2.06	-1.71	-1.21
TMF1	1.03	1.08	-1.89	2.05
TMLHE	-1.65	-2.14	-1.83	-1.17
TMOD2	-1.11	1.07	-2.64	2.82
TMX1	-1.94	-2.53	-1.79	-1.41
TMX4	-2.03	-2.54	-2.33	-1.09
TNFAIP2	1.26	1.62	-1.63	2.64
TNFAIP3	5.73	6.38	2.38	2.69
TNFAIP6	3.76	4.01	2.03	1.97
TNFRSF10B	-1.40	1.12	-2.57	2.87
TNFRSF10D	3.25	3.29	2.22	1.48
TNFRSF9	-3.00	-1.52	-3.76	2.48
TNIP1	2.29	2.94	1.33	2.21
TNPO1	-1.97	-2.24	-2.69	1.20
TNRC6B	-1.34	-2.11	-1.59	-1.32
TOB1	-1.44	-2.08	-1.10	-1.88
TOM1	1.47	2.02	-1.15	2.32
TOM1L2	-1.72	-2.11	-1.64	-1.28
TOP3A	-1.80	-2.14	-1.71	-1.25
TOPBP1	-1.41	-2.61	-1.54	-1.70
TOPORS	1.08	1.26	-2.13	2.68
TP53I11	1.05	-1.65	1.36	-2.24
TP53INP1	-2.27	-2.13	-2.61	1.23
TPP2	-1.84	-2.02	-2.00	-1.01
TPR	-1.12	-1.34	-2.86	2.13
TPRG1L	-2.43	-2.52	-2.38	-1.06
TPST1	-1.64	-1.92	-2.72	1.42
TRAF1	1.87	2.85	1.39	2.05
TRAF3	2.31	2.78	1.40	1.99
TRAK2	-2.22	-2.36	-2.38	1.01
TRAM1	-1.96	-1.82	-2.26	1.25
TRAT1	-1.86	-2.10	-2.10	-1.00
TREM1	-1.08	1.07	-2.46	2.64
TRERF1	-2.11	-2.05	-2.01	-1.02
TRIAP1	-1.23	-1.68	-2.01	1.20
TRIB1	2.31	2.00	2.31	-1.15
TRIM24	-3.08	-3.04	-3.75	1.23
TRIM27	-1.93	-2.39	-2.34	-1.02
TRIM33	-1.82	-2.02	-2.37	1.17
TRIM5	1.76	1.83	-1.22	2.24
TRIM8	-2.08	-2.83	-2.85	1.01
TRIP10	1.75	2.54	1.19	2.14

TRMT112	-1.73	-1.50	-2.59	1.72
TRPM7	-1.38	-1.04	-2.11	2.02
TRPS1	4.10	1.59	6.56	-4.13
TSC22D3	-2.00	-1.58	-2.03	1.29
TSHZ3	-1.42	-2.18	-1.54	-1.42
TSPAN2	-2.22	-2.92	-2.39	-1.22
TST	-2.65	-2.70	-2.38	-1.13
TTC1	-1.37	-1.37	-2.09	1.53
TTL	1.88	2.94	1.09	2.68
TTLL4	-1.10	1.34	-2.09	2.81
TTRAP	-1.96	-1.36	-4.22	3.10
TUBA4A	-1.50	-2.35	1.04	-2.45
TUBD1	2.12	1.44	1.64	-1.14
TUBG1	1.04	-1.20	1.72	-2.06
TUBGCP3	-3.51	-3.40	-3.58	1.05
TUSC4	-2.26	-2.26	-2.31	1.02
TWF2	-2.37	-2.38	-1.91	-1.25
TWSG1	2.08	2.61	1.21	2.16
TXNDC12	-1.82	-1.67	-2.74	1.64
TXNDC3	2.70	1.59	1.22	1.30
TXNRD1	-2.52	-1.84	-3.17	1.72
UBA3	1.09	-1.29	-2.16	1.68
UBA6	2.16	1.14	1.37	-1.20
UBASH3B	-2.14	-1.54	-2.11	1.37
UBE2E1	4.58	5.14	3.06	1.68
UBE2F	2.37	-1.47	1.48	-2.17
UBE2G1	-1.88	-2.27	-2.14	-1.06
UBE2L6	1.13	1.47	-1.43	2.10
UBE4B	-1.86	-2.41	-1.98	-1.22
UBN1	-1.67	-2.03	-1.98	-1.03
UBR1	2.57	1.77	1.85	-1.04
UBR2	-1.48	-1.50	-3.21	2.14
UBR3	-1.54	-2.62	-2.15	-1.22
UBR7	-1.75	-2.07	-1.57	-1.32
UCP2	-1.99	-2.94	-1.12	-2.62
UFD1L	-1.78	-2.02	-2.13	1.05
UGCG	7.06	4.12	5.98	-1.45
UGGT1	-1.52	-1.98	-2.05	1.03
UGP2	2.04	2.50	-1.18	2.96
UHRF1BP1L	-1.50	-1.96	-2.47	1.26
UIMC1	-1.39	-1.75	-2.12	1.21
UNC119	-1.82	-1.68	-2.04	1.22
UNQ1887	-1.74	-1.60	-2.07	1.29
UNQ3104	-1.27	-2.43	-1.21	-2.01
UNQ6228	1.59	2.01	1.02	1.97
UNQ9368	4.05	3.13	1.84	1.70
UPF2	-1.59	-1.96	-2.46	1.25
UPP1	4.45	3.86	4.06	-1.05
UQCRQ	-1.81	-1.72	-2.26	1.32
USF1	-1.24	-1.01	-2.21	2.18

USO1	-1.37	-1.21	-2.08	1.72
USP21	-2.12	-2.24	-1.95	-1.15
USP24	-2.19	-2.28	-2.51	1.10
USP25	-1.35	-1.50	-2.31	1.53
USP33	-1.41	-1.56	-2.18	1.40
USP4	-1.78	-1.60	-2.61	1.63
USP48	-1.47	-2.01	-1.92	-1.05
USPL1	-1.55	-1.19	-2.19	1.84
UTP6	-3.46	-3.30	-3.45	1.05
UVRAG	-1.22	-1.21	-2.07	1.71
VAMP2	-2.33	-2.37	-2.37	1.00
VBP1	-1.51	-2.27	-1.41	-1.61
VCL	-1.30	-2.22	1.06	-2.34
VDAC3	-2.99	-3.55	-3.25	-1.09
VILL	2.63	3.02	1.57	1.93
VIM	-1.13	-1.63	1.23	-2.01
VNN1	3.27	2.09	3.10	-1.48
VNN3	1.05	-1.06	-2.00	1.88
VOPP1	1.91	2.11	1.28	1.65
VPS24	-1.62	-1.58	-2.41	1.52
VPS26A	-1.65	-1.32	-2.06	1.56
VPS26B	-2.29	-2.96	-2.00	-1.48
VPS37C	-1.55	-1.24	-2.16	1.74
VPS41	-1.51	-1.73	-2.48	1.43
VPS8	-3.05	-3.34	-4.72	1.41
VSTM1	1.24	-1.13	2.16	-2.45
WDFY1	-2.11	-1.73	-3.03	1.75
WDFY2	-1.73	-2.04	-1.62	-1.26
WDR26	-1.16	-1.42	-2.18	1.54
WDR47	-2.20	-2.61	-2.13	-1.23
WDR82	-2.36	-1.99	-3.30	1.66
WHSC1L1	-2.73	-3.37	-3.20	-1.05
WIPI1	-1.84	-2.67	-1.12	-2.38
WNT3A	1.22	1.46	-1.41	2.05
WRB	-3.80	-3.65	-2.92	-1.25
WTAP	1.26	1.60	-1.41	2.26
XBP1	4.30	5.41	2.69	2.01
XKR8	-2.20	-2.29	-2.14	-1.07
XPC	-1.84	-1.76	-2.09	1.18
XPO7	-3.10	-3.56	-3.76	1.06
XPOT	-2.09	-2.06	-2.14	1.04
XRCC1	-1.81	-2.20	-1.47	-1.50
XRN1	1.83	1.60	-1.29	2.07
XRN2	-2.23	-2.71	-3.11	1.15
YIPF4	1.45	1.46	-1.46	2.13
YLPM1	-2.00	-2.39	-2.03	-1.18
YPEL2	-1.80	-2.00	-2.21	1.11
YPEL5	-1.62	-1.77	-2.10	1.19
YTHDC1	1.01	1.04	-2.19	2.28
YWHAH	-2.34	-2.39	-1.69	-1.41

YY1	-1.73	-2.04	-1.46	-1.40
ZBTB11	-2.06	-1.60	-1.58	-1.01
ZBTB2	-1.68	-1.31	-2.82	2.15
ZBTB34	-1.75	-1.85	-2.38	1.29
ZC3H11A	1.16	1.30	-1.77	2.30
ZC3H12A	2.45	2.73	1.28	2.14
ZC3HAV1	2.29	2.04	1.28	1.59
ZCCHC2	1.44	1.55	-1.53	2.37
ZCCHC6	-1.18	-1.24	-2.31	1.87
ZDHHC7	-1.82	-2.00	-1.89	-1.06
ZEB1	-2.16	-2.35	-2.49	1.06
ZEB2	1.72	2.25	1.15	1.95
ZFAND5	-1.69	-1.39	-3.14	2.26
ZFP36L2	-2.78	-2.74	-3.39	1.24
ZHX2	2.24	2.76	1.08	2.55
ZMIZ1	-1.78	-1.38	-2.89	2.09
ZMYM2	-1.72	-2.88	-2.21	-1.31
ZMYND8	-1.61	-1.20	-2.25	1.87
ZNF136	-1.43	-1.30	-2.21	1.70
ZNF141	-1.80	-2.04	-2.12	1.04
ZNF146	-2.35	-2.08	-1.91	-1.09
ZNF148	-1.57	-1.74	-2.48	1.43
ZNF18	-2.34	-2.06	-2.95	1.43
ZNF185	-1.82	-1.96	-2.78	1.42
ZNF217	-1.87	-2.43	-2.21	-1.10
ZNF238	-2.00	-2.25	-2.20	-1.02
ZNF250	2.08	1.92	1.34	1.43
ZNF259	2.29	1.56	1.64	-1.05
ZNF266	-1.66	-2.03	-2.08	1.02
ZNF267	1.41	1.64	-1.98	3.25
ZNF277	1.59	1.77	-1.18	2.09
ZNF33A	-1.87	-1.97	-3.07	1.56
ZNF398	-3.39	-3.22	-2.50	-1.29
ZNF417	-1.13	-1.34	-2.15	1.61
ZNF431	-1.35	-2.17	-1.52	-1.43
ZNF480	-2.17	-2.26	-2.31	1.02
ZNF507	-2.38	-2.18	-2.12	-1.03
ZNF552	-1.81	-2.34	-2.12	-1.10
ZNF561	-2.61	-2.60	-2.31	-1.12
ZNF587	-1.74	-2.32	-2.16	-1.07
ZNF592	-1.81	-2.03	-1.66	-1.22
ZNF652	-3.07	-3.59	-3.73	1.04
ZNF673	-1.77	-1.45	-2.01	1.38
ZNF701	-1.59	-1.62	-2.76	1.71
ZNF721	-2.27	-2.50	-2.54	1.02
ZNF828	-1.93	-2.15	-1.58	-1.36
ZNF93	-2.56	-3.00	-2.94	-1.02
ZSCAN16	-1.97	-1.92	-2.53	1.32
ZSCAN22	-2.16	-1.99	-1.64	-1.21
ZXDC	-1.85	-2.07	-1.74	-1.19

ZYG11B	-4.87	-5.95	-4.25	-1.40
